# Innovative gene engineering and drug delivery systems for dendritic cells in cancer immunotherapy

**DOI:** 10.1186/s12929-025-01191-1

**Published:** 2025-11-01

**Authors:** Mridula Prakash, Cedric David Cortez, Akshaya Jayaraman, Sheng-Yun Hsu, Yu-Chi Huang, Chen-Yun Yeh, Yungling Leo Lee

**Affiliations:** 1 Institute of Biomedical Sciences, Academia Sinica, Taipei, Taiwan; 2 Graduate Institute of Life Sciences, College of Biomedical Sciences, National Defense Medical University, Taipei, Taiwan; 3 School of Pharmacy, College of Medicine, National Taiwan University, Taipei, Taiwan; 4 College of Public Health, China Medical University, Taichung, Taiwan; 5 Department of Life Sciences and Institute of Genome Sciences, National Yang Ming Chiao Tung University, Taipei, Taiwan

**Keywords:** Dendritic cells, Genome engineering, CRISPR/Cas9, Nanocarriers, Exosomes, Targeted drug delivery, Cancer immunotherapy

## Abstract

Dendritic cells (DCs) play a crucial role in the coordination of immune responses and have emerged as a potential target for cancer immunotherapy. However, existing DC-based immunotherapies face several clinical challenges, including suboptimal manipulation strategies, poor cross-presentation, and impaired migration. Besides, the complex tumor milieu drives DCs towards a tolerogenic state, leading to immune evasion and cancer progression. Hence, innovative engineering strategies emerging from a thorough understanding of the genetic and molecular aspects of the factors driving DCs to an immune-compromised status will benefit cancer immunotherapy. Taking advantage of the multiplexing potential of gene editing methods such as CRISPR/Cas9 and viral vectors will ensure multiple genome modifications in DCs that can result in higher migration, cross-presentation, and immune-activating cytokine production in a single manipulation step. Such precise DC modifications with high accuracy require the involvement of nanocarrier formulations with high surface functionalization and targeting potential. In this regard, our review provides a comprehensive summary of critical tumor-induced dysfunctions in DCs and promising genome engineering strategies, highlighting nanocarrier-based approaches to mitigate these challenges.

## Introduction

Cancer immunotherapy focuses on activating the immune system and stimulating an antitumor response that may control or completely eradicate tumors [1]. During tumor growth, the complex tumor milieu presents various signaling molecules that significantly impact the development of an effective antitumor immune response. The immunosuppressive nature of the tumor microenvironment (TME) drives immune resistance, becoming the major cited obstacle in effective cancer immunotherapy [2]. The TME accomplishes this by accumulating metabolites and signaling factors, restricting nutrients essential for immune cell function, and limiting immune cell infiltration [3].

Targeting these inhibitory signals restores the tumor-suppressive capacity of the immune system and markedly enhances the efficacy of antitumor immunotherapy [4]. DCs are crucial in dictating the success of cancer immunotherapy. They are the most potent antigen-presenting cells, activating CD4^+^ T helper cells and CD8^+^ cytotoxic T lymphocytes (CTL) to eradicate tumors [5]. However, their efficacy in developing effective antitumor immunity is severely restricted by several tumor-generated factors [5]. Identifying these factors and the molecular pathways by which they negatively regulate DC function provides possibilities for developing strategies that enhance DC therapeutic potential.

In this regard, our review presents a comprehensive analysis of the key tumor-generated factors that induce multiple forms of DC dysfunction in the TME. We further highlight the recent advances in gene editing technologies aimed at engineering DCs to overcome tumor-induced dysfunction and augment their capacity for T cell activation. Lastly, we discuss the latest developments in targeted delivery systems that enable the engineering of DCs for successful cancer immunotherapy.

### DC in cancer immunotherapy

DCs have emerged as the leading candidate for cancer immunotherapy because of their ability to bridge innate and adaptive immunity [6]. These cells orchestrate antitumor responses, and their roles in cancer immunotherapy have been reviewed extensively [7–9]. Immunotherapy exploits DC antigen presentation, migration, cytokine, and chemokine secretion to elicit a robust and sustained systemic antitumor immune response [7].

The most critical functions of DC that are widely explored for tumor immunity are cross-presentation, MHC class II-restricted antigen presentation, cross-dressing, and antigen transfer [10]. DCs internalize tumor-associated antigens (TAAs) from the TME and undergo maturation. This is phase is characterized by the upregulation of co-stimulatory molecules and antigen presentation capacity. Mature DCs migrate from TME to the draining lymph nodes, carrying critical information about the molecular identity and pathogenic potential of tumor antigens [10]. Within DCs, these antigens are processed either through the intracellular cytosolic or vacuolar pathway to cross-present them via endogenous MHC I and elicit TAA-specific CD8^+^ T cell responses [11].

Interestingly, DCs engage in trogocytosis with tumor cells, a term known as cross-dressing [12]. This process involves a transfer of tumor-derived extracellular vesicles (EVs) and intercellular plasma membrane fragments containing intact antigenic peptide-MHC complexes to DCs. This enables direct activation of CD8⁺ T cells without the need for further antigen processing [13]. Additionally, processed TAA peptides suitable for MHC I loading are also transferred from exogenous cells to DCs into TME via gap junctions. This antigen transfer further elicits a robust TAA-specific CD8^+^ T cell tumor response [14, 15].

TAAs processed by lysosomal proteases in endosomal compartments of DCs generate antigen peptides for MHC II loading. These peptide-MHC II complexes are transported to the plasma membrane for presentation to induce CD4^+^ T cell responses. Furthermore, T cell activation is enhanced through the interaction of co-stimulatory molecules CD80/CD86 on DCs with CD28 on T cells [16]. The process of TAA cross-presentation by DCs, including antigen transfer, cross-dressing, co-stimulatory signaling, and cytokine generation, highlights relevant molecular targets and pathways for DC genetic engineering to enhance the significance of cancer immunotherapy.

The potential of DCs can be fully exploited by ex vivo manipulations where these cells are loaded with TAAs and can be used as personalized vaccines that induce aggressive antitumor reactions [17]. Moreover, new treatments involving the active stimulation of DCs to increase the population of T cells specific to tumors are emerging in cancer treatment [18]. Preclinical and clinical trials have shown encouraging outcomes when DC-based immunotherapy is combined with other modalities, such as chemotherapy, radiation treatment, or immune checkpoint inhibition [19].

In both humans and mice, DCs consist of several distinct functional subsets, including monocyte-derived DC (moDC), plasmacytoid DC (pDC), and conventional DCs (cDCs), which are further divided into two types: conventional DC type 1 (cDC1) and type 2 (cDC2) [20, 21]. Comprehensive reviews on the DC ontology and cross-talks between DC subsets are available elsewhere [22–24]. There has been a growing interest in harnessing the potential of various DC subsets for the development of DC-based cancer vaccines in recent years.

#### Traditional and next-generation DC-based vaccines

DC-based therapeutic cancer vaccines overcome the limitations of immune checkpoint blockade (ICBs) and adoptive cell transfer (ACT) therapies, which are caused by cold tumors and an immunosuppressive tumor microenvironment (TME) [25]. Increasing intratumoral abundance of functional DCs and effector T cells is crucial for the success of cancer immunotherapies [25]. A cancer vaccine triggers antigen-specific cellular immunity (CD8^+^ T cells) to eradicate cancer cells. An effective cancer vaccine must improve DC cross-presenting ability for efficient CTL cross-priming, ultimately promoting the success rate of cancer immunotherapy.

In traditional DC-vaccine manufacturing, the CD14^+^ monocytes or CD34^+^ hematopoietic stem and progenitor cells (HSPCs) are obtained from patients via leukapheresis [25]. They are differentiated into immature DCs in the presence of granulocyte–macrophage colony-stimulating factor (GM-CSF) and interleukin 4 (IL-4) and are loaded with TAAs or whole tumor cell lysate along with a maturation cocktail. Finally, the ex vivo manipulated DCs are reinfused into cancer patient for therapy [25]. Due to the easy availability of CD14^+^ monocytes, they are preferentially used in traditional DC vaccines. Sipuleucel-T (Provenge) monotherapy (Phase III trials) and combination therapy with ICB (Phase I and II trials) demonstrated considerable clinical efficiency as an autologous DC vaccine (NCT00065442, NCT01804465, NCT01881867) [26–28].

The efficiency of ex vivo manipulated moDCs to stimulate CTLs is lower than that of the CD34^+^ derived heterogeneous population (NCT00700167, NCT01456104) [25, 29]. However, the restricted numbers of CD34^+^ HSPCs obtained from apheresis limit their clinical application [30]. The need for apheresis, contamination risks, batch-batch variability, DC viability, labor-intensiveness, and cost are significant limitations of this process [25, 31]. Additionally, suboptimal cross-presentation of moDCs, inefficient maturation cocktail, maturation-induced DC exhaustion, poor lymph node homing, and short life span of DC post-injection further restrict the clinical applicability of traditional DC vaccines [31, 32].

Further design of the DC vaccines aims to improve these drawbacks by directly targeting antigens to endogenous DCs in vivo [25]. The antigens are coupled to monoclonal antibodies targeting receptors specifically expressed on the DC surface, such as dendritic and epithelial cells-205 (DEC-205), CD40, or C-type lectin domain family 9 member A (Clec9A) [33]. The requirement of targeted receptor expression on the DC subset and parallel administration of DC maturation factors discourages the clinical implementation of this method [34].

Next-generation DC vaccines focus on designs that can overcome the immunosuppressive events in TME, ensuring an effective antitumor immune response [35]. Ex vivo-generated moDCs are functionally distinct from the steady-state DC subsets present in the body. Thus, DC vaccines incorporating heterogeneous populations of DCs resembling the in vivo condition will benefit the clinical therapy significantly [25, 35]. Next-generation DC vaccines that can potentially activate all DC subpopulations and enhance their interaction with other cells will improve the T cell priming efficacy and vaccine function [35].

##### Biomaterial-based DC vaccine

The biomaterial-based DC vaccines target direct activation of endogenous DCs in situ. Injectable or implantable scaffolds made from biodegradable and biocompatible polymer poly(lactide-co-glycolide) (PLG) offer spatiotemporally controlled release of the loaded cargoes [36]. The scaffold can be loaded with tumor antigens, toll-like receptor (TLR) agonists, adjuvants, GM-CSF, metabolic inhibitors (indoleamine 2,3-dioxygenase 1, IDO1 inhibitor), and various chemoattractants to recruit and activate multiple DC subsets for a prolonged period. Such a vaccine strategy ensures sustained antigen exposure, developing a biomimetic immunogenic microenvironment [37].

Preclinical studies with the B16-F10 melanoma model demonstrated that PLG scaffolds exhibited superior tumor regression within two vaccinations, enhanced IL-12 production, robust CTL infiltration, and minimized Treg accumulation at the vaccination site [38]. The need for multiple scaffold administrations is a limitation observed in this type of DC vaccine. Combining the PLG scaffold DC vaccine with anti-cytotoxic T lymphocyte-associated protein 4 antibodies (CTLA-4) antibodies showed promising tumor regression and CTL activity, preventing multiple scaffold administrations [25, 39]. The requirement for surgical placement of a scaffold, creating discomfort in patients, can be reduced by switching to injectable 3D cryogel systems such as alginate hydrogels [39]. A phase I clinical trial with WDVAX (NCT01753089) for stage IV melanoma is currently in-progress [40]. Further pre-clinical studies and clinical translation evidence are important to validate their efficacy in humans.

##### Immunogenic cell death (ICD)-inducing DC vaccine

ICD-inducing DC vaccines transform cold tumors to hot tumors by enhancing antigenicity and adjuvanticity [41]. The release of tumor antigens and exposure to danger signals such as high mobility group box 1 (HMGB1), calreticulin, heat shock protein 70 (HSP70), and adenosine triphosphate (ATP), enhance DC recruitment and T cell priming [41, 42]. The ICD-released antigens and damage-associated molecular patterns (DAMPs) activate DCs, inducing their homing to tumor-draining lymph nodes (TdLNs) to cross-prime T cells. Chemotherapy, photodynamic therapy (PDT), photothermal therapy (PTT), and radiotherapy are various ICD triggering methods [42, 43]. Combining ICD-based DC vaccine with other immunotherapies has been shown to enhance the antitumor efficacy in both preclinical and clinical studies by activating tolerogenic DCs to immunostimulatory DCs, enhancing cross-presentation ability, and increasing intratumoral infiltration of CD8^+^ T cells. Such multimodal strategies have shown delayed metastasis, an 80% systemic tumor remission rate, and prolonged patient survival. Accessibility of the tumor sites, toxicity concerns, and limited systemic effects are important concerns to be addressed for successful clinical application of ICD-inducing DC vaccine.

##### mRNA-pulsed DC vaccine

Traditional DC vaccines often face challenges such as human leukocyte antigen (HLA) restriction, poor antigen presentation, and suboptimal effector T cell induction. To circumvent this, pulsing DCs with tumor mRNA has emerged as a promising technique [44]. This strategy activates TLR-mediated immune responses, offers engineering feasibility, and represents a complete antigen repertoire, reducing immune escape. mRNAs are delivered via electroporation into DC cytosol, preventing their degradation and ensuring intact DC function [45].

The need for large tumor samples and the presence of inhibitory molecules limit the applicability of tumor-derived mRNAs. To overcome this, in vitro transcribed mRNAs have been designed. This allows modification to improve stability and efficacy. To enhance their therapeutic efficacy, strategies such as fusion with trafficking sequences or lysosomal sorting signals will promote antigen presentation and effector T cell responses [46]. Preclinical and clinical studies have reported significant enhancement in DC activation and tumor-specific T cell priming upon co-transfection of DCs with antigenic and immunostimulatory molecules mRNAs such as CD83, OX40L, 4-1BBL, cluster of differentiation 40 ligand (CD40L), and glucocorticoid-induced tumor necrosis factor receptor (GITR). Similarly, co-transfection with stimulatory cytokine mRNAs such as IL-15, IL-12, and GM-CSF has also shown promising pre-clinical efficacy [47, 48].

In advanced solid tumors, intratumoral delivery of anti-programmed death ligand 1 (PD-L1) (durvalumab) and interleukin 12 (IL-12) mRNA is currently undergoing a Phase I clinical trial. Combining mRNA pulsed DC vaccines with ICB is another strategy that has proven durable tumor eradication clinically [49]. The method of administration is crucial in determining the effectiveness of mRNA therapy. Current strategies focus on investigating various in vivo mRNA delivery methods, including intratumoral, intranodal, intradermal, and intravenous injections.

##### DC-derived small extracellular vesicles (DCsEVs) vaccine

DCsEVs are stable, retain critical immunostimulatory functions, prime CD8^+^ T cells, carry high levels of CD80/CD86, and antigen-loaded MHC-I/MHC-II complexes [50]. The first generation of DCsEVs was designed with peptide-loaded moDCs but exhibited insufficient cross-presentation and poor T cell activation. The second generation of DCsEVs was produced from IFN-γ matured moDCs and showed superior immunostimulatory potential. However, due to their poor in vivo distribution and presentation, the activation of antigen-specific T cell responses was significantly restricted. The third generation of DCsEVs explored the usage of other subsets of DCs [25, 50].

The clinical success of pDC vaccination in inducing CD8 + T cell responses has led to the design of pDCsEVs. This strategy exploits the ability of pDCs to transfer antigens to cDCs, thus combining the strength of pDCs and cDCs in eradicating tumors. Further extensive validations are necessary to evaluate the clinical efficacy of pDCsEVs in cross-presentation [51]. Another strategy to enhance the clinical efficacy of DCsEVs is by combining ICB with DCsEVs. For instance, anti-CTLA-4 modified DCsEVs and anti-PD-1 engineered DCsEVs have shown promising preclinical efficiency in eradicating tumors, inducing DC migration to TdLNs, and CTL activation compared to ICB monotherapy [52].

Despite their success in tumor suppression, there are certain challenges that need to be addressed. Their precise location and characterization are critical as DCsEVs are heterogeneous and can be contaminated by sEVs from off-target cells. Careful understanding of EV biology, trafficking, tumor interactions, and molecular engineering must be given importance for unlocking the complete potential of DCsEVs in cancer immunotherapy [25, 50, 53].

**Fig. 1:**
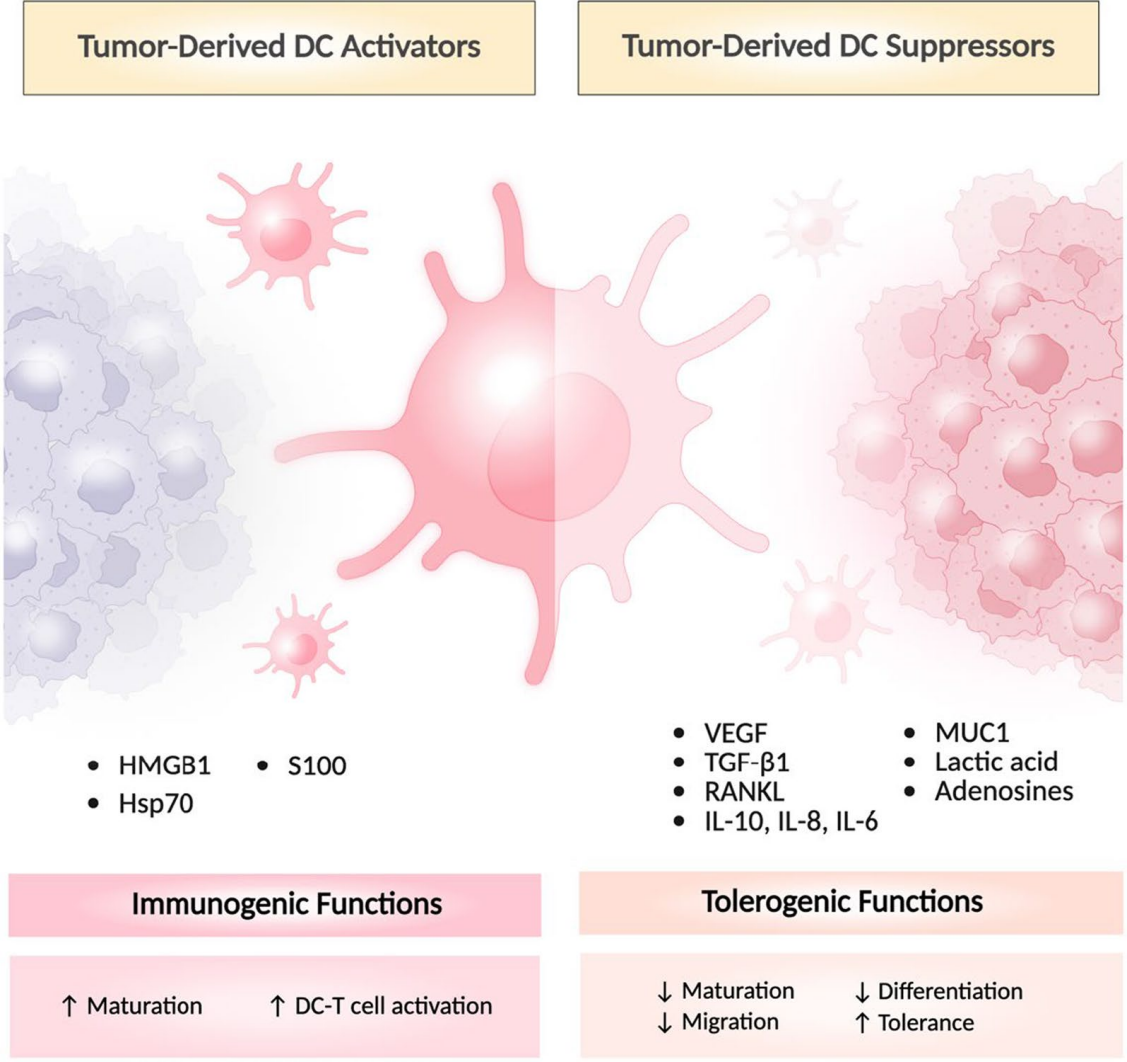
Tumor-derived factors shape DC function in the TME. Tumor-released HMGB1, Hsp70, and S100 promote immunogenic functions in DCs. The anti-inflammatory cytokines, lactic acid, VEGF, and MUC1 from tumors suppress DC differentiation, maturation, and migration, promoting DC tolerance, leading to tumor progression. Figure illustrated with BioRender

Despite the developments made in DC-based vaccines, their therapeutic efficacy is compromised due to immunosuppressive TME (Fig. 1). The complex tumor milieu limits DC infiltration, maturation, activation, migration to the tumor-draining lymph node (TdLN), cross-presentation, and promotes tolerance to suppress the activation of CTL and foster tumor growth [54]. In addition, repeated TAA exposure in the TME also drives DC into a functionally exhausted state that further inhibits CTL activation [55]. Hence, for developing effective cancer immunotherapy, identifying these causes and effective preventive approaches is critical. In the following section, we discuss the various ways by which TME induces DC dysfunction in mounting an effective antitumor immunity to suppress cancer.

### TME-induced dysfunction of DC

The peripheral tissue resident DCs are the primary responders to tumor antigens in situ. In a functional state, these DCs migrate to the TdLNs to activate T cells and B cells mediated immune responses [56]. cDC1s are the predominant DC subsets in cross priming and activating CD8^+^ T cells, whereas cDC2s activate CD4^+^ T cell responses. However, once the tumor growth begins in a tissue, the resident DCs encounter multiple challenges that reprogram their antitumor abilities. Under such circumstances, the tissue resident DCs often promote immune suppression and tumor progression rather than initiating antitumor immunity [56].

The secondary lymphoid organs comprise both migratory DCs from TME carrying antigens and LN resident DCs that capture soluble and transferred antigens from lymph and blood. Synergistically, they prime naïve T cells, differentiating them to effector T cells and B cells equipped for eradicating tumors [7, 57]. The functioning of DCs in the secondary lymphoid tissues is also significantly altered during tumor development. This prevents effective T cell priming and encourages the expansion of regulatory T cells (Tregs). Rather than serving as regions of potential antitumor activity, such TdLNs transform into immunosuppressive niches promoting tumor progression [7, 58, 59].

**Fig. 2:**
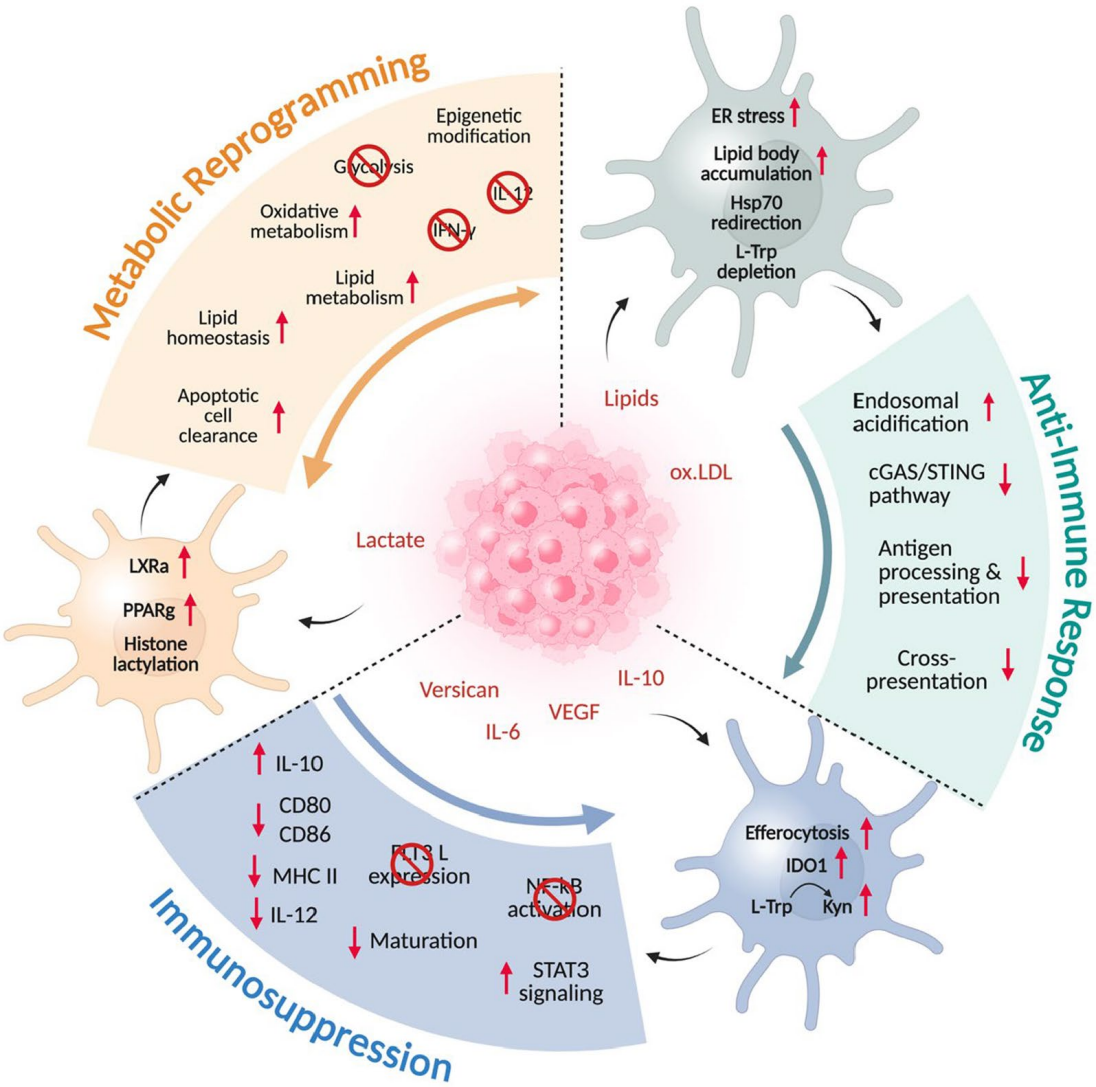
TME induced dysfunction in DC. The tumor-derived factors induce metabolic reprogramming in DC, promoting DC exhaustion in the TME. The metabolic shift redirects DC to immunosuppression and an anti-immune phenotype, resulting in tumor progression. Figure illustrated with BioRender

The infiltration of DCs into the TME is a major challenge faced in cancer therapy. Although natural killer (NK) cells within the tumor generate chemoattractants for effective tumor infiltration of DCs, the beta-catenin signaling in tumor cells inhibits DC recruitment into the TME [60, 61]. Upon infiltration, DCs have to overcome multiple challenges generated by the TME to remain functionally active and to induce potent antitumor activity. Tumor-derived factors negatively regulate DC maturation, activation, migration, and cross-presentation ability to suppress their antitumor function [61]. In this section, we categorize the TME-induced DC dysfunctions based on their metabolic reprogramming, anti-immune response, and immunosuppression (Fig. 2).

#### Metabolic reprogramming

Within the TME, the fate of DCs towards activation or tolerance solely depends on DC metabolism [62]. The metabolic events in DC are closely associated with its maturation. The pro- and anti-inflammatory phenotypes in DC are regulated by the adenosine monophosphate (AMP)-activated protein kinase (AMPK)/ mechanistic target of rapamycin (mTOR)-mediated switch between oxidative phosphorylation and glycolysis [63, 64]. Upon TLR stimulation, the AMPK signaling activates the oxidative phosphorylation (OXPHOS) while inhibiting the glycolytic pathway and mTOR signaling in DC. This significantly suppresses the co-stimulatory molecule expression, maturation, and T cell activation ability of DCs, directly resulting in tumor progression [63]. In the TME, DCs often encounter a high lactate, nutrient-depleted, and hypoxic environment. The lactate uptake and hypoxic circumstances cause robust glycolysis in DC without sufficient mitochondrial OXPHOS support. This leads to a dysfunctional state in DC, which ultimately inhibits T cell activation. Hence, for effective DC function in TME, a fine balance must be created between glycolysis for rapid energy production and OXPHOS for sustained antigen processing and presentation [64, 65].

Hypoxia in the TME affects DC metabolism, decreasing their maturation and antigen presentation ability [66]. The reduced oxygen levels within TME also impact T cell migration and effector functions, substantially weakening the antitumor immune response [67]. Hypoxia coupled with abnormal hypoxia-inducible factor 1α (HIF-1α) activity increases glycolysis and accumulation of lactate, which results in acidosis [68]. Such metabolic changes impede DC maturation processes by blocking type I interferon (IFN-I) signaling, which is required for effective DC activation [68]. Marquez et al. reported the mechanism by which hypoxia disrupts the regulation of HIF1α, and their role in downregulating the glycolytic gene expression required for efficient DC activation [69]. Furthermore, mTORC1 suppression dampens cytokine signaling and compromises T cell stimulation [70–72].

Another important factor is the increased adenosine concentration in the TME. This promotes DC differentiation into CD209-expressing subsets that possess altered functional properties and promote immune tolerance, aiding in tumor progression [73]. In the TME, lactate and pyruvate accumulation induce HIF1α protein, suppressing DC maturation and cytokine production [74]. DCs acquire a chronic inflammatory status within the TME, accompanied by reduced CD80/CD86 expression, diminished IL-12 production, and limited migratory ability. Under such circumstances, prolonged antigen exposure causes DCs to adopt an anti-inflammatory phenotype [75]. Tumor-induced inflammation causes DC exhaustion via the NF-kB and STAT3 signaling pathways, reducing their ability to initiate an antitumor immune response [76].

Fatty acid metabolism is yet another critical metabolic event in DC that affects its activation and maturation. Tumor-derived immunosuppressive factors enhance fatty acid oxidation (FAO) and OXPHOS, driving excessive lipid uptake and lipid peroxidation in DCs [73, 77, 78]. This shift in metabolism suppresses pro-inflammatory cytokine secretion and antigen cross-presentation, aiding immune evasion [73]. The intracellular lipid accumulation in DC has been identified as a major cause of their diminished cross-presentation potential in TME. Upon DC uptake, the oxidized lipids (ox lipids) inactivate the chaperon protein, HSP70, by directly binding to it intracellularly, thereby preventing HSP70 interaction with lysosomes [79]. This results in MHC accumulation in the lysosomes, preventing their presence on the cell surface, blocking the tumor antigen presentation to T cells. The high lactate present in tumor-infiltrating DCs (TIDCs) also decreases the endosomal pH, which promotes excessive antigen degradation. This suppresses its antigen presentation and blocks IL-12 and IFN-γ generation [80].

Another factor is the downregulation of autophagy-related protein Atg5 and the concurrent upregulation of CD36 and CPT1 molecules in DCs [73]. Increased LPO within DCs causes functional impairment and promotes tumor activity via a mechanistic link to the activation of the inositol-requiring enzyme 1 α-x-box binding protein 1 (IRE1α-XBP1) pathway, along with the accumulation of lipid peroxides. Treatments focused on reducing lipid peroxides have been effective in restoring DC activity and lessening immune suppression within the TME [81].

The ER stress resulting from altered lipid metabolism in TME is known to reprogram TIDC into a tolerogenic phenotype [77, 82]. Notably, the absence of the adaptor protein BAT3 in DCs enhances a tolerogenic phenotype and induces a hyperactive unfolded protein response (UPR), promoting T cell immunoglobulin and mucin domain-containing protein 3 (Tim-3) dominance and impairing antitumor T cell activity [83]. In tumor settings, mechanisms such as unfolded protein accumulation in DCs trigger ER stress through the IRE1α-XBP1 signaling axis, ultimately impairing their antigen-presenting capacity [78, 84].

In addition, the engulfment of apoptotic tumor cells rich in cholesterol and lipids activates peroxisome proliferator activator receptor gamma (PPARγ) and liver X receptor alpha (LXRα) in TIDCs [85]. This further reprograms metabolic events in DCs by promoting lipid uptake and accumulation, decreasing glycolysis, suppressing the NF-kB pathway, and promoting anti-inflammatory cytokine generation [86]. Collectively, this results in failure of DC maturation and antigen presentation, thereby skewing towards DC exhaustion in TME [86]. As a result, DCs are unable to potentiate a robust antitumor immune response mediated by T cells due to the intense metabolic reprogramming induced by the tumor factors.

L-tryptophan in DC is essential for T cell proliferation, differentiation, and effector activities. Tryptophan depletion significantly impairs T cell antitumor response [87]. TME damages the DC-T antitumor interaction by depleting L-tryptophan reserves in DC. The inhibitory interaction between CTLA-4 in T cells and CD80/CD86 in DCs upregulates IDO1 expression in DCs [88]. IDO1 is the key enzyme responsible for converting L-tryptophan to kynurenine, thereby activating a cascade of anti-immune events downstream [88]. Kynurenine-mediated anti-immune response in DC is an emerging field of interest that needs more attention to elucidate several target genes in DCs that contribute to antitumor response. Enhanced IDO-1 expression has been reported in TIDCs, and IDO-1^+^ DCs suppressed CD8^+^ T cell activation, proliferation, and effector function, while enhancing Treg differentiation [89].

In addition to TIDCs, DCs present in the TdLNs are also metabolically rewired by the tumor to prevent effector T cell priming and promote tumor growth. The tumor-derived factors, such as lactate, adenosine, exosomes, VEGF, and prostaglandin E2 (PGE2), reach the TdLNs through afferent lymphatics [90]. This upregulates the scavenger receptor CD36 on CD8α^+^ DCs to increase the lipid uptake, thereby impairing antigen processing and presentation [91, 92]. The accumulation of lipids inhibits glycolytic flux, induces mitochondrial dysfunction, causes oxidative stress, enhances IDO-1 expression, and skews DCs in the TdLNs towards a tolerogenic phenotype [91]. This collectively suppresses the IL-12 production and T cell priming ability of DCs in TdLNs [92]. Additionally, the suppressive cells from the TME, including tumor-associated macrophages (TAMs) and myeloid-derived suppressive cells (MDSCs), also migrate to the TdLNs [93]. They secrete arginase-1, which depletes arginine in DCs, creating an amino acid starvation status that results in tolerogenic DCs [94, 95]. The tumor released factors and vesicles also reach the spleen via the bloodstream. Similar to the TdLNs, these factors also suppress glycolysis, enhance lipid uptake, and promote immunosuppressive events in splenic DCs. MDSCs from TME also accumulate in the spleen, which further promotes amino acid starvation, tryptophan depletion, mitochondrial dysfunction, and altered lipid metabolism in splenic DCs [96]. Cumulatively, these events lead to impaired DC maturation and activation, inducing Tregs rather than effector T cells, thereby causing systemic immunosuppression.

In summary, the immunomodulatory factors present in the TME trigger metabolic reprogramming in DCs, leading to various immunosuppressive effects, ultimately resulting in an anti-immune phenotype. Identifying the key genes and molecular pathways in DC that are amenable to immunosuppressive factors in the TME ensures successful DC modification to improve cancer immunotherapy. For instance, blocking lactate influx into DC and inhibiting lactate signaling is one strategy to overcome lactate-induced suppression of DC maturation. Targeting inhibition of CD36 receptors on CD8α^+^ DC also assists in preventing lipid-mediated changes in Batf3^+^ DCs. Modifying the HIF-1α pathway to prevent hypoxia-driven glycolytic dominance in DC is another suitable strategy. Maintaining healthy mitochondrial biogenesis and choosing the right DC subset for therapeutic manipulation will ensure an immune-responsive DC population in the TME.

#### Anti-immune response

##### Inhibition of cross-presentation

The cross-presentation ability of TIDCs, LN resident and migratory DCs, is significantly impaired by the metabolic changes occurring in the TME. The oxidative stress and lipid-enriched environment in tumors causes lipid peroxidation and the generation of by-products such as 4-hydroxynonenal (4HNE) and malondialdehyde (MDA). [81]. Once taken up by TIDCs, the lipid by-products interfere with protein folding in the endoplasmic reticulum (ER), accumulating misfolded proteins. This triggers ER stress response and activates the unfolded protein response sensor, inositol-requiring enzyme 1 α (IRE1α), leading to the transcriptional activation of XBP1 [97]. In Batf3^+^ CD8α^+^ cDC1, XBP1 upregulates the genes involved in lipogenesis, thereby causing lipid accumulation within the cell, disrupting antigen processing, presentation, MHC I/II surface expression, and co-stimulatory molecule expression. As a result, the lipid-stressed cDC1 in TME significantly decreases the CTL activation and promotes tumor immune evasion [79].

Moreover, aerobic glycolysis-driven generation of lactic acid by the tumor cells accumulates in the TME. These lactic acids enter the moDCs through monocarboxylate transporters (MCTs) and inhibit moDC differentiation into functional DCs [86]. They also impair p38 MAPK and NF-kB signaling pathways, inhibiting cytokine production and moDC maturation. Furthermore, lactic acid in moDCs stabilizes HIF-1α, preventing antigen uptake, processing, and presentation [86]. Cumulatively, these events prevent CTL priming and the mounting of an effective antitumor immune response.

##### Inhibition of DC migration

The tumor-released factors also hinder the migration of DCs from TME to the tumor-draining lymph node (TdLN), thus preventing the CD8^+^ T cell priming. The tumor secretes several oxysterols, namely 22(R) hydrocholesterol or 27-hydrocholesterol, that diffuse into the TME and bind to liver x receptor-α (LxRα) present on DCs [98]. LxRα is a transcription factor, and its ligand activation results in nuclear translocation and transcriptional repression of CCR7 gene activation and DC migration [99, 100]. TAMs, B regulatory cells (Bregs), and MDSCs in the TME and TdLNs secrete IL-10 and TGF-β that downregulate CCR7 expression on DCs and suppress their migratory potential [93]. In addition, these immune cells generate arginase, ROS, and modify the ECM in the TME via matrix metalloproteinases (MMPs) to prevent DC migration [95]. Additionally, the remodelled ECM and the suppressive cytokines VEGF, PGE2, IL-10, and TGF-β, generated by TAMs, Tregs, Bregs, and MDSCs, also prevent DC infiltration into the TME [94, 95].

##### Inhibition of cGAS/STING signaling in DC

In addition, TME suppresses DC activation, antigen presentation, and the cytotoxic T lymphocyte (CTL) mediated antitumor response by hindering the activation of the cGAS/STING pathway in DCs. This pathway promotes DC maturation, activation, TAA cross-presentation, and effector T cell responses [101]. Immunogenic cell death (ICD) releases various DAMPs, such as ATP, HMGB1, and calreticulin, which are sensed by DCs [102]. The released HMGB1 recruits nucleic acids from dead tumor cells into the endosome of DCs, initiating innate nucleic acid sensing [103]. This results in the production of interferons (IFNs) and other inflammatory cytokines by activating the cGAS/STING pathway, which induces DC activation and efficient antigen presentation to prime an effector T cell response against tumor cells [103]. The tumor cells evade the immune response by preventing innate nucleic acid sensing in DCs. The T cell immunoglobulin mucin receptor 3 (TIM3), expressed on tumor-infiltrating cDCs, competes with tumor-derived DNA for binding to HMGB1 [104]. The sequestration of HMGB1 prevents its binding with tumor nucleic acids, inhibiting DNA sensing and DC activation, thereby promoting tumor escape [104].

Furthermore, the tumor cells dampen antitumor response by engaging in the CD47-SIRPα interaction. The CD47 present in tumor cells interacts with SIRPa receptors in cDC2 in the TME [105]. In addition to preventing phagocytosis by DCs, the CD47-SIRPa interaction inhibits the ability of cDC2 to internalize and initiate an immune response to mtDNA released by the tumor cells. Their interaction leads to the recruitment of tyrosine phosphatase, SHP1, to the phagosomal membrane to inactivate NADPH oxidase, NOX2, and enhance phagosomal acidification-mediated mtDNA degradation [106]. This further prevents cGAS/STING pathway activation, type I IFN production, and CTL activation, thereby blunting the antitumor immune response [107].

In a nutshell, the TME-derived factors reduce the activation, maturation, migration, and cross-presentation ability of TIDCs and TdLN DCs, leading to the suppression of anti-immune response. By focusing on certain strategies, these challenges can be mitigated. For instance, targeted delivery of STING agonists to Batf3^+^ cDC1s, inhibiting WEE1 kinases and cGAMP-degrading enzyme, ENPP1, can ensure activation of the cGAS/STING pathway, promoting DC activation and maturation [108]. The STING agonist STINGVAX has recently emerged as a powerful adjuvant in cancer immunotherapy [109]. In a B16 melanoma mouse model, STINGVAX, consisting of cyclic dinucleotides (CDNs) plus GM-CSF, significantly increased CD8^+^IFNγ^+^ T cell and DC infiltration into the tumors [110, 111]. To promote TIDCs’ migration to TdLNs, strategies to enhance the expression of CCR5 and CCR7 in cross-presenting DC subsets, inhibit adhesion molecules such as E-cadherin, prevent transcriptional repression of CCR7, and enhance the interaction of CCR7^+^ DC subsets with CCL21^+^ lymphatic endothelial cells could be promising. Inhibiting LxRα receptors on DCs to prevent oxysterol uptake is also a suitable option.

Increasing the expression of GSTA4 and ALDH2 could potentially inhibit the uptake and effects of 4-HNE and MDA on DC cross-presentation. Investigating the specific receptors and transporters involved in 4-HNE and MDA uptake by DC also yields promising therapeutic outcomes. In addition, blocking IRE1α nuclease, XBP1 transcriptional activity, XBP1 protein translation, and GRP43 helps in preventing UPR response, preventing lipid accumulation, thereby rescuing antigen processing. Using MCT inhibitors to block lactate transporters in moDC is another strategy. Inhibiting HIF1α stabilization and inhibiting the pathways influencing HIF1α activity, such as PI3K/AKT/mTOR and MAPK, is another suitable strategy.

#### Immunosuppression

TME releases several immunosuppressive factors that prevent DC infiltration and negatively affect the antitumor response of DCs. An abundance of cDC1 population within TME is positively correlated with high responsiveness to immune checkpoint inhibitor (ICI) therapy in cancer patients [60, 112]. Several cancers are reported to have a lower population of cDC1 in the TME due to their decreased infiltration, viability, and differentiation [27]. One strategy by which the tumor cells evade immune response is by secreting intrinsic factors that affect cDC1 migration into the TME. In human and mouse tumor models, beta-catenin-positive tumors suppress CCL4 expression to prevent cDC1 migration into the TME and promote tumor growth [113].

The tumor cells also influence other immune cells to negatively regulate the recruitment of cDC1 to TME. NK cells are the major producers of CCL5, XCL1, and FLT3L in the TME that promote cDC1 infiltration [60]. Tumor cells suppress NK cells’ viability and chemokine secretion via PGE2 to restrict cDC1 infiltration and prevent T cell activation in the TME to promote tumor growth [114]. Moreover, the *in-situ* development, survival, and proliferation of cDC1 depend predominantly on FLT3L in TME. The tumor cells produce several factors, such as VEGF, IL-6, and PGE2, to inhibit FLT3L activity and prevent cDC1, moDC, and pre-DC maturation, differentiation, and survival in the TME, causing an immunosuppressive environment for tumor progression [115, 116]. To address this challenge, utilizing selective COX2 inhibitors along with blocking IL-6 and VEGF is a promising approach.

In addition, the other immune cells present in the TME create a constant immunosuppressive environment that can influence the functional status of DCs. For instance, the TAMs with M2 phenotype and Bregs attract T regulatory cells (Tregs) into the TME [94]. Tregs engage in interaction with TIDCs via the CTLA-4-CD80/CD86 axis, thereby upregulating IDO-1 in DCs, resulting in a dysfunctional phenotype [95].

The immunosuppressive TME also disrupts the CTL-activating cytokine production in DCs (Fig. 1). They release versican, which acts as an agonist for TLR2 in DCs [117]. This interaction promotes the production of IL-6 and IL-10 in DCs and leads to an overexpression of IL-6 and IL-10 receptors to facilitate hyperphosphorylation and activation of the STAT3 signaling cascade, to dampen IL-12 generation in DCs [117, 118]. Blocking IL-6R/IL-10R and inhibiting versican-TLR2 interaction can prevent the progression of immunosuppressive DCs. Blocking TLR2 using an anti-TLR2 antibody in DC has been shown to inhibit MMP2-induced cytokine production and OX40L upregulation. In addition, designing peptides that block versican interaction with TLR2, silencing versican synthesis in tumors, and blocking HA to prevent the versican-HA-TLR2 complex are other powerful methods to overcome immunosuppression.

In TME, DCs undergo efferocytosis, a process where DCs recognize the apoptotic cancer cells and engulf them to prevent an inflammatory reaction [85]. The engulfed cancer cells are degraded in the phagolysosomes, resulting in the upregulation of receptor tyrosine kinases (RTKs) and the release of immunosuppressive cytokines IL-10 and TGF-β [119–121]. This immunosuppressive polarization event prevents DC maturation and activation [121]. The IL-10 and TGF-β present in the TME directly hinder the development and function of DCs by reducing the levels of CD80 and CD86 that are needed to activate T cells [122]. These signals downregulate MHC class I and II molecules on DCs, thereby impairing their ability to effectively prime CD8⁺ and CD4⁺ T cells. This impairment fosters T cell exhaustion driven by the interaction of PD-1-PD-L1 between T cells and DCs [4]. Disrupting the immunosuppressive effects of TME on DCs represents a novel strategy to improve the effectiveness of cancer immunotherapy.

The accumulation of immunosuppressive factors and signals in TME enhances the expression of inhibitory receptors, PD-L1, VISTA, BTLA, and LAG3 on DCs [123]. The dysfunction of DC is marked by the upregulation of these immune checkpoint molecules, which impair maturation, antigen presentation, and cytokine secretion in DCs, resulting in diminished T cell activation [124, 125]. Combining immune checkpoint inhibitors with other immunotherapies has been shown to be successful.

Furthermore, the degree of DC exhaustion may serve as a predictive biomarker for immunotherapy response, emphasizing the importance of focusing on this phenomenon to improve treatment efficacy. For instance, to overcome DC exhaustion in the TME, approaches must aim to enhance DC function by promoting their maturation to effectively activate CTLs. Strategies using TLR and STING agonists have been investigated to reverse the exhaustive state of DCs toward an immunogenic phenotype in vivo [126, 127]. However, their poor pharmacokinetics, suboptimal stability, low bioavailability, transient activation, rapid clearance, and constant immunosuppressive TME demands for superior therapeutic approaches targeting DC maturation and activation in TME that can activate robust T cell immunity [128, 129].

Therefore, approaches to overcome the metabolic reprogramming, anti-immune effects, and immunosuppression in DCs present in TME and TdLNs are critical in the success of DC-based cancer immunotherapy. In the recent past, genome engineering has emerged with tremendous potential to perform permanent gene modification in immune cells to suppress tumor growth. DC reprogramming in the TME involves the activation of specific signaling pathways and transcription factors that regulate the synthesis of immunostimulatory and immunoregulatory molecules. In the following section, we present the recent developments in genome engineering techniques for improving DC function (Table 1).

**Table 1:** A comparison of different genome engineering strategies in DC

Features	Viral vector	TALEN	CRISPR	Refs.
Suitable DC subsets	• Suitable for primary DC subsets (cDCs and pDCs) where electroporation is difficult	• Suitable for iPSC manipulation	• Suitable for moDCs and BMDC manipulation where electroporation is feasible	[163, 322–324]
Design	• Easy design • Vector type dependent • Laborious vector constriction for transduction	• Complex design • New protein pair engineering needed for each target	• Easy design and gRNA sequence dependent • Difficult to perform in primary DC subsets due to electroporation	[325]
Targeting	• Gene insertion at random and predefined sites • Can integrate into “safe harbors”	• No PAM requirement • Any sequence can be targeted	• Restricted by PAM requirement	[326]
Specificity	• Unpredictable integration sites	• Very high specificity • Protein-DNA recognition • Suitable for targeting immune regulatory genes in DC • Suitable for immunogenic DC reprograming	• High specificity with possible off-target effects	[324]
Off-target effect	• Risk of insertional mutagenesis	• Lesser off-targets effects	• Possible off-target effects due to gRNA mismatch • Cause of concern while editing immune modulators in DCs	[327]
Efficiency	• Highly efficient in primary DC subsets cDCs and pDCs • Viral integration ensures long term expression of target genes in DC subsets	• Efficiency depends on cell type • Lower efficiency in primary DCs	• Highly efficient in MoDCs and BMDCs • Electroporation of RNPs feasible • Less efficient in cDCs and pDCs	[162, 163]
Multiplexing	• Restricted possibility to co delivery of multiple viral constructs due to packaging limits	• Difficult to edit multiple genes simultaneously • Large protein requirement	• Possible to edit multiple immune regulators simultaneously with various gRNAs • Possible to knock out of IL-10 and PD-L1 in MoDCs and BMDCs to overcome immunosuppression	[322–324, 326–329]
Versatility	• Highly versatile	• Moderate versatility	• Highly versatile	[330]
Delivery	• Easy delivery; effective in vivo and ex vivo • Transduction favourable for cDCs and pDCs	• Harder to package and deliver efficiently due to larger proteins	• Small gRNAs and Cas9 nuclease can fit in viral/non-viral systems • Electroporation favourable for MoDCs and BMDCs • Unfavourable for cDCs and pDCs	[331, 332]
In vivo application suitability	• Well established for in vivo applications • Several FDA approved clinical trials use vectors	• Delivery challenges makes it less suitable	• Highly promising • Delivery strategy needs further research particularly in cDC1s, cDC2s and pDCs	[329]
Safety	• Size restrictions, risks of immune responses and insertional mutagenesis	• Lower risk of off-target effects • Possible to cause double stranded breaks	• Possible off-target effects, p53 activation and large deletions • Risk of auto immune response due to dysregulated immune pathways in DCs	[140–143]
Suitability	• Suitable for permanent gene expression, stable gene addition, editing machinery delivery and in vivo therapy • Most suitable for cDC1s, cDC2s and pDCs using transduction	• Suitable for high specificity editing of difficult targets and clinical cell therapies • Less practical for DC engineering due to complex design and delivery requirements	• Suitable for functional genomics and high throughput editing • Suitable for ex vivo manipulation of BMDCs and MoDCs using electroporation	[326]
Possible DC engineering for immunotherapy	• Overexpression of soluble cytokines IL-12, GM-CSF, and TAA • In vivo DC reprograming	• Precise editing of mono immunoregulatory genes in DC • Tolerant DC generation (HLA-DR deficient DC) • DC mitochondrial gene manipulation • Suitable for DC vaccine generation	• Immunostimulation • Immune check point inhibition • Migration and maturation • Co-stimulation • Cross-presentation • Reprograming DC function using CRISPRa/i	[143, 154]

## Genome editing strategies for DC engineering

Genome editing has recently become a widely used technology for manipulating DC functions to enhance clinical outcomes in cancer immunotherapy [130]. Several gene editing platforms, including viral vector-based, RNA interference (RNAi), zinc finger nucleases (ZFNs), transcription activator-like effector nucleases (TALENs), and clustered regularly interspaced short palindromic repeats (CRISPR)-Cas-associated nucleases, have been successfully employed to modify various immune cells, such as natural killer (NK) cells and T cells [130]. A comparison of various features of the most commonly employed gene editing techniques for DCs has been detailed in Table 1. However, advanced gene editing technologies to engineer DCs for improved antitumor responses and to address dysfunctions induced by the TME are still relatively underexplored.

**Fig. 3:**
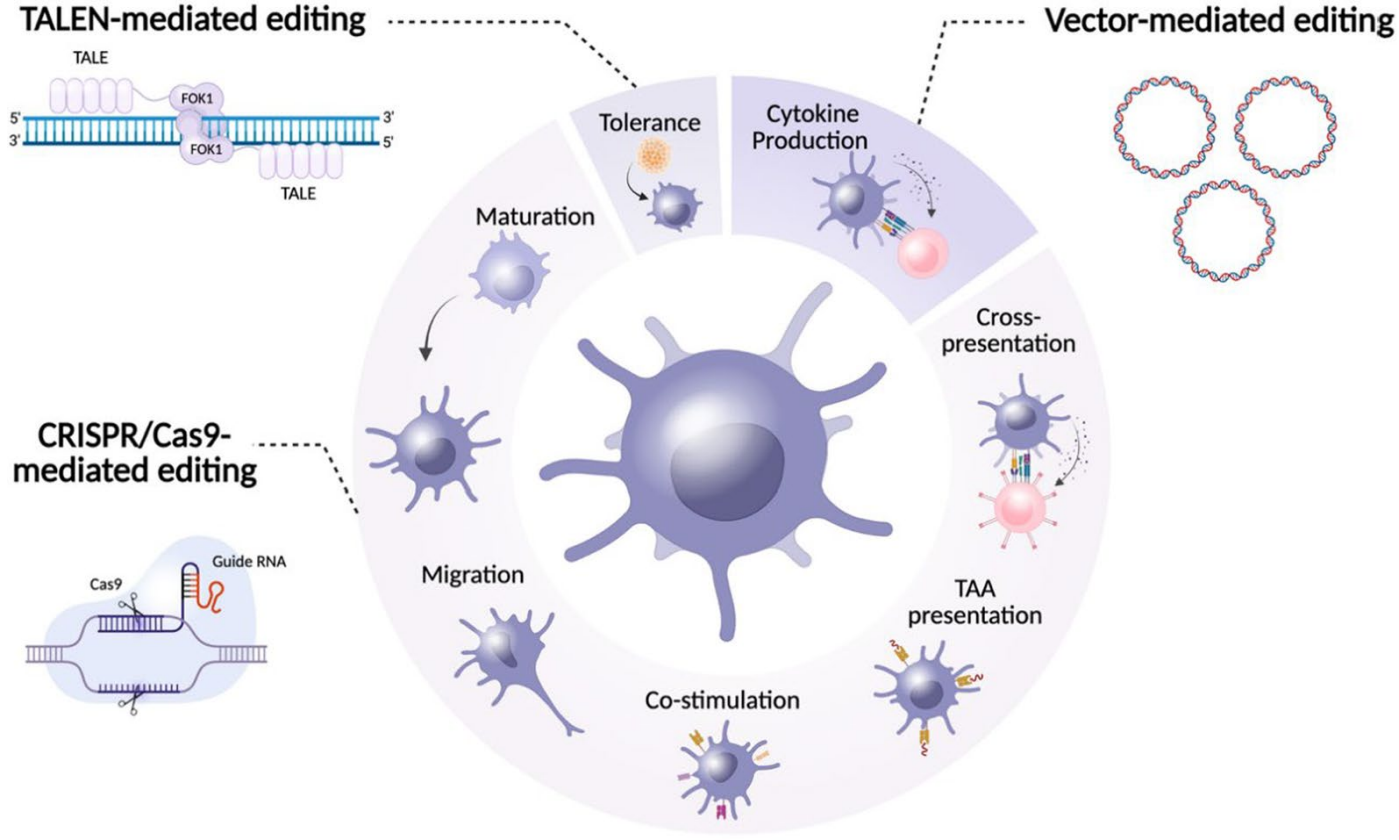
Genome editing strategies used in DC manipulation

A significant amount of research has concentrated on CRISPR/Cas9-mediated editing of T cells, particularly focusing on targeting PD-1 to boost antitumor activity and reduce T cell exhaustion caused by PD-1/PD-L1 interactions [131]. Given that PD-L1 expression is also a key immune checkpoint in DC-T cell interactions and plays a role in T cell exhaustion, there is considerable interest in investigating the most effective gene editing approaches to modulate this relationship. Among the various methods available (Fig. 3), the genetic reprogramming of DCs using CRISPR/Cas9 genome editing is particularly noteworthy. Genome editing of DCs can be applied at multiple stages of the T cell activation process, including major histocompatibility complex (MHC) class I and II antigen presentation, co-stimulation, maturation, migration, and evading immune suppression, all to enhance their antitumor responses [132, 133]. Several efforts have been made to identify suitable DC subsets for genome manipulation [134–137]. In the following sections, we will review recent advancements in DC genome editing, emphasizing its applications in cancer immunotherapy.

The majority of DC gene editing approaches focus on CRISPR/Cas9 technology, followed by strategies involving viral vectors and TALEN. Figure illustrated with BioRender.

### CRISPR/Cas9-mediated gene editing

**Fig. 4:**
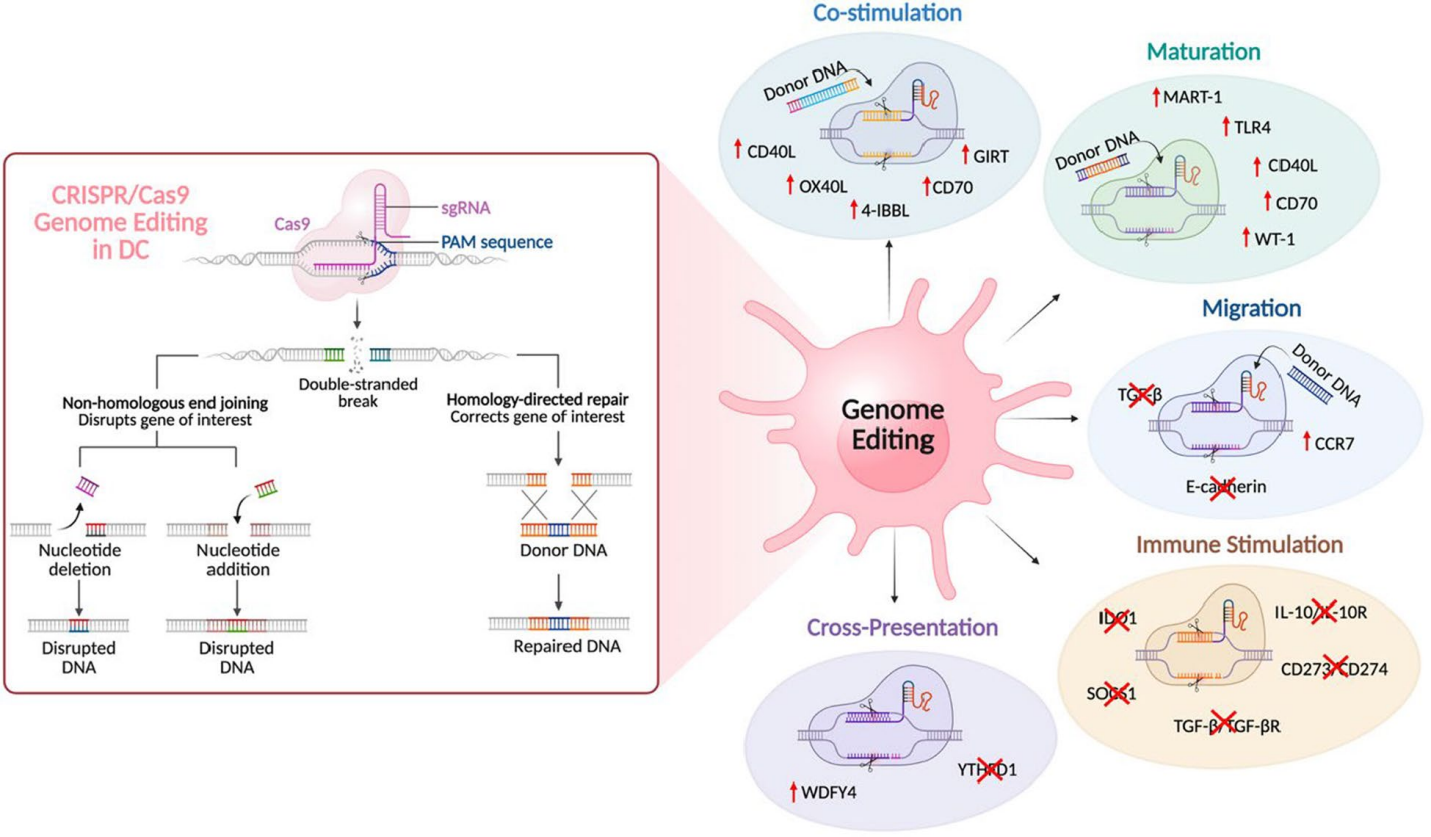
Promising CRISPR/Cas9 genome editing approaches in DC. Efficient gene knock-in and knock-out can be attained via Cas9-mediated homology-directed repair and non-homologous end joining in DC. CRISPR/Cas9-activated modification in the DC genome can boost its antitumor potential by promoting the expression of co-stimulatory molecules, cross-presenting genes, maturation, and migration inducers. The knockout of immunosuppressive genes in DC helps to overcome TME-induced dysfunctions and inhibit tumor progression. Figure illustrated with BioRender

CRISPR/Cas9-mediated genetic knockouts in DCs offer a powerful strategy to systematically knock out gene targets that regulate key aspects of DC biology. This approach enables the discovery of genes essential for DC-tumor interaction, migration, and maturation into effective antigen-presenting cells (Fig. 4) (Table 2). Genetic manipulation of human DCs has faced challenges due to limited efficiency and specificity [138, 139]. However, the advent of CRISPR/Cas9 technology has enabled the construction of targeted genetic knockouts in human blood-derived moDCs with greater than 94% efficiency across 300 genes in response to TLR 4 stimulation. This approach successfully recapitulated the well-established TLR signaling pathways, highlighting key target genes involved in ligand recognition and downstream immune responses [140]. As a result, hematologic malignancy patients, such as those with multiple myeloma, lymphoma, or leukemia, undergoing HSPC transplantations significantly benefit from CRISPR/Cas9 genome editing of DC due to improved T cell activation [135, 141].

**Table 2:** Summary of CRISPR/Cas9 genome engineering in DC

CRISPR/Cas9 delivery methods	Target DC	Target Genes	Purpose	In vitro studies	In vivo studies	Refs.
Cas9 transgenic mice	Murine CD8α ^+ ^/XCR1 ^+ ^cDC1 and moDCs	WDFY4	• Screening • Identify XPT regulators	• ↓ T cell proliferation	• ↓ Cross-presentation • ↓ Tumor regression	[154]
Cas9-RNP electroporation	Human moDCs	PTPN6/ SHP-1	• Screening • Identify TLR4 stimulators	• TNF-a and IL-10 secretion study	-	[140]
Lipid NP delivery of Cas9 mRNA	cDCs	PD-L1	• PD-L1 knockout	• ↑ Cas9 mRNA uptake • ↑ PD-L1 knockout	• CT26 colon cancer model • ↓ PD-L1^+^ cDC1 in dLN and TME • ↑ CD40, CD80 & CD86 in cDC1 • ↑ Tumor suppression	[143]
Cationic lipid NP delivery of Cas9 mRNA + gCD40	BMDC	CD40	• CD40 knockout	–	• Acute mouse skin transplant model • ↑ Transplant tolerance • ↓ T cell activation & graft damage	[157]
Mx-Cas9-GFP transgenic mice	HOXB8 MPP	IRF8	• IRF8 knockout	–	• ↓ IRF8 promoter • ↑ DC differentiation	[160]
Cas9-RNP electroporation	Murine myeloid lineage cells	MyD88	• MyD88 knockout	• ↑ IL-12 production	• MyD88 significant in cDC1 mediated host immunity	[161]
Cas9-RNP nucleofection	BMDC	NDRG2	• NDRG2 knockout	• ↓ DC maturation • ↑ Regeneration	• Diabetic mouse model • ↑ wound healing	[162]
Cas9 transgenic mice	BM-DC	CCR7 GCaMP6S	• Multiple editing in Cas9-HOXB8 cell	• CCR7 importance in DC migration	• CCR7 significant for DC migration	[162]
Cas9 transgenic mice Cas9-RNP electroporation	Murine & Human HSPC	Eed, Suz12, DNM3TA	• Multiple editing in HSPC	• 60 and 75% gene knock out in HSPC • > 20% human HSPCs with homology directed repair	–	[142]
dCas9-VPR-mRNA or CD5_CRISPRa nucleofection	Human skin DC2 Cord blood derived CD34^+^ DC Human moDCs	CD5	• ↑ CD5 in DC2 & moDC • CD5 knockout	• ↑ T cell proliferation • ↑ IFNɣ, IL-13 & TNFα production • ↓ T cell proliferation	• MCA1956 and MC38 tumor model • CD5^+^ DC critical for T cell immunity • ↑ CD5^+^ DC in ICB therapy	[163]
Cas9 transgenic mouse	Murine BMDC	PAF & OST complex	• Screening • Identify TNFα producers	–	–	[155]

#### Immunostimulation

Within the TME, DCs are continually exposed to immunosuppressive signals mediated by small molecules, cytokines, and apoptotic tumor cells. CRISPR/Cas9-mediated genome editing engineer DCs to resist these suppressive influences and enhance their immunostimulatory function [132]. Gundry and team reported efficient ex vivo knockout of genes in CD34^+^ HSPCs-derived DCs by CRISPR/Cas9 editing. The Cas9-gRNA ribonucleoprotein complex (RNP) was electroporated to generate efficient knockouts of multiple genes without affecting the DC viability and proliferation. This strategy could effectively silence single or multiple immunosuppressive genes such as PD-L1/PD-L2, IL-10, IL-10R, TGF-β, TGF-βR, SOCS1, or IDO in DCs ex vivo to improve their antitumor response [142].

#### Immune checkpoint molecules

The tumor cells succeed in suppressing effector T cell functions in TME by engaging in immune-deficient receptor-ligand interactions with T cells. The interaction of PD-1 on T cells with their ligand, PD-L1, results in dysfunctional CD8^+^ T cells driving their exhaustion. CRISPR/Cas9-dependent knock-out of PD1 on CD8^+^ T cells ex vivo and PD-L1 on multiple tumor cells has been successfully established [131]. Knockout of PD-L1 expression in cross-presenting DCs, such as cDC1s, presents a promising therapeutic intervention in maintaining the cytotoxic function of T cells. Recently, Mao and team developed a lipid-based delivery system to execute the knock-out of PD-L1 in CD11c^+^ MHCII^+^ XCR1^+^ CD11b^−^ cDC1 and CD11c^+^ MHCII^+^ XCR1^−^ CD11b^+^ cDC2 in vivo*.* The delivery of Cas9 mRNA and PD-L1 gRNA in cDCs resulted in enhanced XCR1^+^ cDC1 activation, cross-presentation, T cell stimulation, and suppression of colon cancer growth in a murine model [143]

#### Maturation

The CRISPR/Cas9 also allows gene editing via homology-directed repair [144]. For instance, the expression cassette with TAA cDNA can be introduced for integration into the gene editing target region, allowing TAA's constitutive expression. The endogenously expressed TAAs will be processed and loaded onto MHC I/II for CD4^+^/CD8^+^ T cell activation. This strategy could also be employed for the constitutive expression of DC maturation-inducing genes TLR4, CD40L, and CD70 by ex vivo manipulation of moDCs during vaccine generation. CRISPR/Cas9-mediated gene expression enables the use of mature DCs within hours of gene delivery, compared to the traditional method that requires 24 h of incubation in a maturation-inducing mix, causing DC exhaustion [135, 142].

#### Migration

Migration of DCs from the TME to the lymph nodes plays a key role in antitumor immune response. The interaction of C–C motif chemokine receptor 7 (CCR7) with its ligand chemokine C–C motif ligand 21 (CCL21) drives the migration and accumulation of cross-presenting DCs in the lymph nodes, leading to T cell priming and tumor rejection [145]. CRISPR/Cas9 could be an effective tool for upregulating the expression of CCR7 in DCs [110]. Employing CRISPR/Cas9-mediated targeted knockout of genes involved in DC tissue retention could be an effective alternative for enhancing their migration [146]. The ex vivo engineering of CD11c^+^ DCs by CRISPR/Cas9-mediated knock out of the homing factor E-cadherin, its positive regulator TGF-β, and LxRα receptor potentially increases the expression of CCR7 in DCs, resulting in enhanced migration [147].

#### Co-stimulation

CRISPR/Cas9-mediated gene insertion could boost the co-stimulatory function of DCs [110]. The major co-stimulatory interactions between DC and T cells for antitumor immunity include CD80/CD86/CD28, CD40/CD40L, 4-IBB/4-IBBL, OX40/OX40L, GIRT/GIRTL, and CD27/CD70 [10, 47, 148–150]. Thus, strategic ex vivo manipulation of CD11c^+^ DCs using CRISPR/Cas9 guided expression of CD40L, OX40L, 4-IBBL, GIRT, and CD70 ensures autonomous maturation and licensing of DCs, leading to effective CTL activity without a TAA-specific T cell response.

#### Cross-presentation

CRISPR/Cas9-based editing can influence genes associated with cross-presentation to enhance the CTL activity [151]. Excessive antigen degradation in DCs is a major cause for diminished CTL activation. For instance, in mice, YTHDF1 protein promotes the translation of lysosomal cathepsins involved in antigen degradation, thereby suppressing cross-presentation [152]. In vivo CRISPR/Cas9-guided knockout of ﻿*Ythdf1* improves cross-presentation and antitumor response mediated by Batf3^+^ cDC1 in mice. The initial investigation on the relevance of YTHDF1 in human colon cancer biopsies indicated that low levels of YTHDF1 correlated with high CTL abundance [152]. Cross-presentation heavily depends on endosomal vesicles and their trafficking network for the efficient intracellular transport of antigens. This is critical for antigen processing, MHC class I loading, and transport back to the plasma membrane for T cell presentation [153]. Identifying the key vesicular trafficking genes could be an efficient way to improve cross-presentation and tumor suppression. CRISPR/Cas9 screening of ex vivo differentiated cDC1s and primary splenic cDC1s identified WDFY4 as critical for cross-presentation [154]. The absence of *Wdfy4* in an immunogenic fibrosarcoma mouse model showed a significant failure in tumor regression. WDFY4 was found to be associated with protein assembly and endosomal vesicular trafficking within the cell [154]. This emphasizes the importance of a CRISPR/Cas9-based genome-wide screening of moDCs or CD34^+^ HSPC-derived DCs to identify potential genes involved in controlling the cross-presentation in DCs.

#### TLR activation

Bone marrow-derived DCs present another suitable platform for genome-wide CRISPR/Cas9 screening to study the complex innate immune circuits activated upon receptor stimulation [155]. The oxidized LDLs and extracellular matrix fragments in TME act as DAMPs that activate TLR 4 in TIDCs. The initial activation of TLR 4 in TIDC results in pro-inflammatory cytokines, including TNFs. However, their chronic activation in TME results in negative feedback loops and metabolic reprogramming in DC, thereby driving them to an immunosuppressive state [156]. Thus, TLR 4 activation in TME and identifying their downstream signaling in DC presents a wide therapeutic opportunity to maintain mature DC in an antitumor state. Parnas et al. identified the significance of the platelet-activating factor (PAF) complex and the oligosaccharyltransferase (OST) complex in inducing tumor necrosis factor (TNF) production upon TLR4 stimulation of BMDC using CRISPR/Cas9 genome-wide screening [155]. This identification of novel PAF and OST’s role in the downstream signaling of TLR 4 activation in CD11c^+^ MHCII^+^ DC could be a therapeutic target for maintaining the antitumor status of DC in TME.

#### Transplant tolerance

In addition to enhancing the antitumor immune response in DC, CRISPR/Cas9 genome editing has been used to promote tissue transplant tolerance in DC by reprogramming CD40 expression [157]. The off-target cleavage site generation in the genome is a major concern associated with CRISPR/Cas9 gene editing in DCs [158]. A study conducted by Fu and team showed that CRISPR/Cas9 modification can cause significant off-target gene editing at sites that differ by five nucleotides from the target sequences, promoting the chances of tumorigenesis in highly proliferative cells [158]. This can be avoided by using Cas9-gRNA RNP complexes rather than other methods for Cas9 delivery. Additionally, mutating Cas9 to reduce the DNA binding energy while maintaining its effect on-target ensures CRISPR/Cas9 safety [159].

##### CRISPRa

CRISPR gene activation approach (CRISPRa) using dead Cas9 (dCas9) fused to transcriptional activators is a promising technique to induce the expression of endogenous genes without DNA modification [160]. CRISPRa has been successfully employed for upregulating CD5 expression in human and mouse DCs without any adverse effect on their T cell activation potential [160]. DCs also play a critical role in the recruitment of CD8 + T cells into the TME and their reactivation. In particular, cDC1 secreted CXCL9 and CXCL10 are the major chemokines that recruit CD8 + T cells into the TME [129, 130, 161]. CRISPRa targeting cDC1 to upregulate the expression of CXCL9 and CXCL10 could be an efficient way to improve the infiltration of CD8 + T cells in the TME. Future research must focus on multiplexed editing using CRISPR/Cas9 to simultaneously manipulate several signaling pathways, including IL-12 secretion and CCR7 migratory receptor expression, to induce a robust immune response against the tumor.

### TALEN-mediated gene editing

TALEN is another prominent gene-editing tool used to edit specific DNA sequences by inducing a double-stranded break in DNA. Its subsequent DNA repair introduces mutations, inserts DNA sequences, or deletes existing sequences, leading to gene silencing [162]. Kwon et al*.* performed genome editing using TALEN to generate a universally compatible immune nonresponsive human induced pluripotent stem cell (iPSC). By knocking out HLA-DR in iPSCs, the various cells developed, including DCs, did not express HLA-DR. Importantly, the knockout of HLA-DR in DCs did not induce a strong CD4^+^ T cell response, and such technology could be advantageous in cases with a shortage of donors [163].

TALEN is a remarkable gene editing tool with a robust targeting capacity of unlimited sequences. They are suitable therapeutic platforms for human genome modifications with low cellular toxicity, high specificity, and precision [164]. Although TALEN-based gene editing is well studied in creating CAR-T cells, PD-1 knockout in T cells and multiple cancer cells, its application in DC gene editing, especially for cancer immunotherapy, has not been widely explored compared to CRISPR/Cas9, mainly due to its complex engineering and assembly requirements [164]. Nevertheless, TALEN offers high specificity and low cell toxicity while editing across diverse cell types. Unlike CRISPR, TALEN exhibits minimal off-target effects [165]. This is particularly critical when targeting a specific gene or an allele for editing. TALEN offers methyl sensitivity over CRISPR by detecting methyl modifications of cysteine residues [165]. TALEN can also target mitochondria for modifications, which is still a challenge in the case of CRISPR/Cas9. Mito-TALEN (mitochondrial-targeted TALEN) has been proven efficient in studying human mitochondrial diseases caused by mitochondrial DNA mutations [166]. Hence, Mito-TALEN can be a suitable strategy to understand the role of mtDNA in improving DCs’ antitumor function.

### Vector-mediated gene editing

The vector-mediated gene editing involves the use of viral vectors such as AAVs, lentivirus, and adenovirus to deliver editing tools. The vector itself can act as a main editing tool, such as transposons or integrating viral vectors, independent of CRISPR and TALEN editing. In comparison to CRISPR and TALEN, the vector-mediated editing approach offers certain advantages. They offer higher delivery efficacy due to the high transduction efficacy in primary cells, HSCs, and neurons, while CRISPR and TALEN often rely on direct RNP delivery or electroporation, significantly affecting cell viability [167, 168]. The integration of the viral vector can provide a permanent expression of the target gene. On the other hand, CRISPR and TALEN often provide a one-time incision and depend on the cells’ intrinsic repair pathways, which can be challenging in the case of non-dividing cells. In addition, the viral vectors offer precise gene additions. They can insert a full-length gene without depending on HDR, whereas CRISPR and TALEN face HDR limitations. While CRISPR-based knockouts are very effective, their ability for precise knock-ins is less efficient [168, 169].

Unlike CRISPR and TALENs, Viral vector-mediated systems avoid genotoxic risks by avoiding double-stranded breaks. This avoids undesired deletions, P53 activations, and translocations [168, 170]. Viral vectors provide cargo versatility, whereas CRISPR and TALEN require additional delivery systems for cargoes such as donor DNA templates. In addition, the viral vector-based systems offer promising therapeutic translations. Various FDA-approved late-stage clinical therapies use AAVs and lentiviral vectors for in vivo delivery [171]. Although CRISPR and TALEN are powerful ex vivo manipulation techniques, their successful in vivo delivery applications still remain pre-clinical. Viral vector-mediated engineering of DCs has proven to be highly efficient in various applications [172].

Cytokines secreted by cDCs are known to play an important role in mediating an immune response [173]. For example, IFN-g and IL-12 secreted by cDCs are essential for the maturation and activation of CD8^+^ T cells [174]. However, the cytokines secreted by cDCs are minimal and are subjected to their surrounding microenvironment [175]. This, in turn, sparked an interest in overexpressing cytokines in DCs to modulate immune response.

Overexpression of soluble cytokines in DCs using DNA and adenoviral vector vaccines has been attempted in animal models. Among the cytokines, granulocyte–macrophage colony-stimulating factor (GM-CSF) overexpression has gained particular interest as it plays a crucial role in DC survival. The transduced DCs overexpressing GM-CSF matured with strong CTL priming ability [176, 177]. Another cytokine of interest is Fms-like tyrosine kinase 3 ligand (Flt3L), as it is essential for cDCs development. In a murine model, DCs transduced to overexpress Flt3L lead to increased cDC1 and control of tumor growth [178].

While soluble cytokines secreted by transduced DCs were effective in animal models, a human trial with IL-12 secreting transduced tumor-infiltrating lymphocytes led to cellular toxicities attributed to the secreted IL-12 [179]. A proposed solution was the overexpression of cytokines on DC membranes, which is achieved by generating a plasmid with a fused cytokine and transmembrane domain [180]. The added advantage of this system is its ability to act on specific cell types in a juxtacrine manner [181]. For instance, CD8^+^ T cell stimulation and Th1 polarization were increased when DCs were transduced with membrane-bound IL-12.

[182]. Hence, DC functions can be modulated using plasmid transduction, positively regulating the cancer survival outcome. Lentiviral vectors (LVs) have been employed to edit the DC genome for differentiating into cDC1s [183]. For instance, melanoma-specific tumor antigen, tyrosin-related protein 2 (TRP2), expressing cDC1s was generated by introducing a lentiviral vector encoding TRP2-GM-CSF and IL-4 to patient-derived CD14^+^ monocytes [184]. Similarly, LVs encoding for FLT3L and Notch signaling have shown significant potential to differentiate HSPCs to pDCs and cDC1s.

In all, we showed that DC functions are malleable, while transient DC editing methods have the advantage of being reversible, they come with the downside of being susceptible to changes in the microenvironment. On the other hand, permanent DC editing methods could withstand tolerogenic conditions generated by the tumor microenvironment. However, there is a risk of creating other unforeseen side effects as each gene in dendritic cells has a role to play.

For effective antitumor response, DCs must overcome the immunosuppressive environment induced by TME. Although there are advanced gene editing techniques to modify DC function, their efficiency solely depends on the efficient delivery of genome editing tools into DC. CRISPR-based gene editing offers multiple advantages over other editing tools; however, it must be used in combination with suitable viral vectors to achieve maximum clinical success. In the following sections, we discuss the recent developments in various drug delivery platforms targeting DC for cancer immunotherapy.

## Nanotechnology and targeted delivery systems for DC

Novel targeted delivery systems focusing on cancer immunotherapy have received prominence in recent years due to high efficiency, targeting specificity, and minimized off-target effects [185]. While existing therapeutics target cancer cells [186–188], newer strategies in nanoparticles (NP) formulation over the decade emphasized the role of nanocarriers in targeting antigen-presenting cells (APCs), educating them to combat tumor cells [189]. The interaction between MHC class I/II in DCs with TCR in CD4^+^/CD8^+^ T cells is critical in initiating an active antigen-specific antitumor immune response. As a result, recent studies on nanoformulations have focused on strategies to boost DC-T interaction for initiating potent antitumor effects [190–192].

Previously, DC-based immunotherapies relied on ex vivo DC maturation from peripheral blood monocytes (PBMCs). To induce maturation, immature DCs are pulsed with tumor antigens and reintroduced into patients. This approach is labour-intensive and has limited in vivo efficacy and persistence [188, 193, 194]. Interestingly, next-generation DC therapies using NPs offer promising outcomes that enhance therapeutics via antigen loading, direct in vivo interactions with immature DCs, reproducibility, and sustainability [188]. In addition, nanocarriers offer prolonged circulation, effective cell targeting, and surface functionalization to evade clearance by the mononuclear phagocytic systems [195, 196]. Hence, NP formulation is a crucial factor that decides the efficiency of nanocarriers in DC-based cancer immunotherapies (Table 3). Several platforms, such as liposomes, polymeric micelles, and albumin-based therapies authorized for cancer treatment, are currently being developed [187, 195, 197].

**Table 3:** Examples of nanocarriers used for DC targeting in cancer immunotherapy

Nanocarrier		Classification	Cargo	Target in DC	Route of delivery	Cancer type	Clinical significance	References
Organic NP	Lipid NP	LNP	OVA *Trp2* mRNA HPV E7 mRNA	MYD88/RLR-independent STING pathway	*i.m.* or *s.c.* injection	B16-OVA melanoma	• Antigen-specific mRNA vaccination	[206]
		LNP	uniSTING mRNA	STING IRF3/IFN-I cascade	*i.t.* or systemic injection	4T1 B16-F10 Hepa1-6 ES-2 THP-1 LLC1	• DC maturation • Antigen-specific CD8^+^ T-cell responses	[211]
		RNA-lipoplexes	viral/mutant neo-antigens endogenous self-antigens	IFNα	*i.v.* injection	B16-OVA, CT26, TC-1	• Effector T cell & tumor mRNA expansion • RNA immunotherapy	[333]
		Empty LNP (eLNP)	-	IRF7 CD40	In vitro uptake	-	• T_FH_ priming • DC maturation	[207]
		Empty ionizable LNPs	-	TLR4 NF-κB IRF	In vitro uptake	-	• LNP design for specific signaling pathways	[334]
		Lipid NP	BLANmCas9/ gPD-L1	PD-L1	Transfection	Colon cancer CT26	• Gene editing • cDC1 activation • T cell stimulation	[142]
	Polymeric NP	Acid-degradable polymeric NP	Ovalbumin	DEC-205	Vaccination (s.c.)	–	• ↑ In vivo CTL activation	[335]
		Biodegradable poly(lactic-co-glycolic acid) (PLGA)	Ag, Pam3CSK4 and Poly(I:C); agonistic αCD40-mAb (NP-CD40) coating	CD40	Vaccination (s.c.)	B16-OVA	• ↑ DC maturation	[139]
		PEG-*b*-PLGA-based cationic lipid-assisted nanoparticles (CLAN)	mCas9, sgRNA_CD40_	CD40	Transfection (in vitro), *i.v.* (in vivo)	-–	• Transplant tolerance • Graft survival	[156]
Inorganic NP	Silica	Silica-layered calcium PEGylated Ca(OH)2/SiO_2_ core–shell NP	αCD205/DEC205 coating	DEC205	In vitro/ex vivo co-culture/ cellular uptake; *i.t* (in vivo)	B16F10-OVA, B16F10, MB49	• ↑ CD40 & MHC-II • ↑ Ag presentation • ↑ DC migration	[194]
		Dendrimer-like PEI-grafted silica NP	OVA_257-264_-αCLEC9A conjugate 2′3′-cGAMP STING agonists	CLEC9A, STING pathway	*s.c.* & *i.v*	B16-OVA	• STING-mediated Ag cross-presentation • T cell activation • Artificial APC • DC maturation	[209]
		Mesoporous silica MSNP-MaN-PDL1bp/CLT	PD-L1 binding peptide (PDL1bp), clotrimazole (CLT)	Mannose receptor (CD206), PD-1/PD-L1, CD80/CD28, and MHC II/T cell receptor	*s.c*	B16-F10	• ↑ MHC II-mediated Ag presentation • PD-L1 blockade	[193]
		Multifunctional MSN	TLR7 agonist (R848)	TLR7	In vitro uptake (culture)	–	• Non-toxic & non-inflammagenic drug delivery	[336]
		Extra-Large Pore MSNs	OVA, CpG oligonucleotide (TLR9 agonist)	TLR9	*s.c*	B16-OVA	• Protein/adjuvant co-delivery • ↑ DC activation	[337]
		MSN	OVA	-	*s.c*	B16F10-OVA	• ↑ Ag cross-presentation	[338]
		Hollow MSN	OVA, Poly(I:C) (TLR3 agonist), αPD-L1 Ab	TLR3	*s.c*	EG7-OVA	• ↑DC activation • ↑Ag cross-presentation ICB dose minimization • ↑CD4^+^/CD8^+^ T cell responses	[339]
		MSN@TheraVac	HMGN1, R848, αPD-L1 Ab	TLR7	*i.v*	CT26	• Tumor-infiltrating DC maturation • ↓ T cell exhaustion	[340]
		DMSN	OVA, CpG oligonucleotides	TLR9	*s.c*	B16-OVA	• Tunable pore size • ↑Ag loading • Controlled release of OVA and CpG	[341]
	Gold NP (AuNP)	10 nm or 50 nm (AuNP_10_ and AuNP_50_)	–	TLR2 TLR4	In vitro uptake	HEp-2	• Impaired DC maturation • T_H_2 and T_H_17 polarization	[239]
		< 2–12 nm AuNPs and NCs	–	CD80 CD86	In vitro uptake	–	• ↑ DC maturation • ↑ IL-12, IFN-γ, & IL-10 • ↑ T & NK cell proliferation	[342]
	Iron dot/oxide NP	RNA-loaded magnetic NP	RNA	–	Ex vivo transfection *i.d.* injection	–	• ↑ DC migration	[225]
		Iron oxide–zinc oxide (Fe_3_O_4–_ZnO core–shell) NP	ZnO-binding peptides -carcinoembryonic antigen	–	In vitro uptake, immunization	MC38, CEA	• CEA-specific immune responses • ↑ IFN-γ-secretion	[343]
		Iron oxide (IO) NPs	mRNA-encoding tumor Ag	–	Vaccination	B16F10-OVA	• ↑ IFN-α production • ↑ DC migration	[344]
		Magnetic Fe_3_O_4_@Ca/MnCO_3_	OVA	Mn^2+^-induced STING pathway, Ca^2+^-induced autophagy	*s.c*	OVA antigen	• Cytoplasmic delivery • ↑ CD8^+^ T cells & Ab levels	[226]

*s.c.*—subcutaneous, *i.v.*—intravenous, *i.t.*—intratumoral, *i.d.*—intradermal, *i.m.*—intramuscular

LNPs, for instance, when conjugated with ligand specific to DCs allow reduced hepatic uptake. To date, most LNP formulations available in the market tend to localize mostly in the liver among other organs [198]. As an example, the first Food and Drug Administration (FDA)-approved LNP siRNA drug Patisiran (ONPATTRO^®^) accumulated in the liver. LNPs formulated with similar ionizable lipids (i.e., SM-102, cKK-E12, and C12–200) also showed unintended liver tropism [199–201]. While these NPs offer efficient and feasible mRNA cargo encapsulation, non-hepatic delivery remains an issue with their use. Since the goal of NP DC-based immunotherapies is to deliver cargo in DCs, successful administration to secondary lymphoid organs (SLO) such as the lymph node and the spleen is a must.

Other formulation focuses on stealth strategies, including PEGylation and incorporation of immune-evasive ligands [170]. For instance, the addition of an endogenous albumin-binding lipophilic tail and a polyethylene glycol (PEG) block linked in subunit vaccines (termed CpG-DNA/peptide amph-vaccines) reduced systemic toxicity and increased antitumor efficacy of resident phagocytes [202]. These innovations in nanocarrier designs are significant in instances where an efficient and precise in situ delivery of antigens, immunopotentiators, and genome editing tools is required without inducing cytotoxicity for DC engineering.

**Fig. 5:**
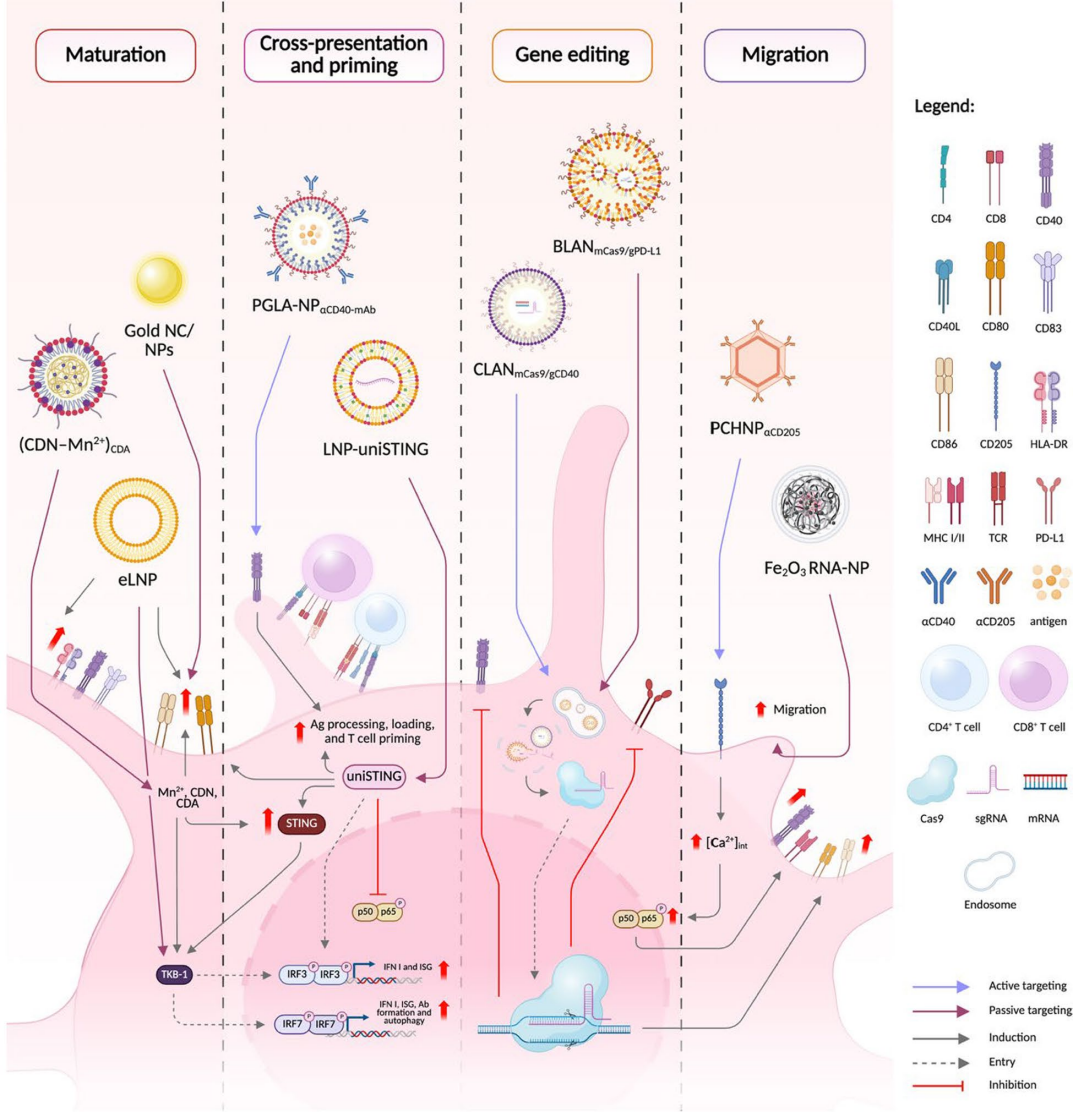
Mechanism employed by nanocarriers to engineer DC in cancer immunotherapy. Nanoparticles enter DC via passive mechanisms and receptor-mediated endocytosis. They enhance DC maturation, migration, and cross-presentation via intracellular calcium and STING pathway activation. Nanoparticles carrying Cas9 or Cas9/gRNA RNP complexes perform genetic engineering in DC to induce antitumor abilities. Figure illustrated with BioRender

In this regard, the following section discusses NP formulations aimed at improving DC-mediated anti-cancer response. We also highlight various NP-induced intracellular mechanisms in DCs to boost DC-T interaction (Fig. 5) and how each NPs offers unique advantages and limitations **(**Table 4**)**. Moreover, this section enlightens the nanocarriers developed so far for introducing CRISPR/Cas9 systems for DC engineering.

**Table 4:** Advantages, opportunities, and limitations of commonly used nanoparticles in DC targeting

Nanoparticle	DC targeting strategies	Cargo	Comparison to Other NPs		
			Advantages and opportunities	Limitations	Refs.
Lipid-based NPs	Passive targeting to mannose and DEC-205 receptors	Nucleic acids Antigens Adjuvants Cas9 RNP	• Clinically validated • Cargo protection from degradation • Minimal cell toxicity and scalability	• Lipid components can trigger innate responses • Formulation-reliant biodistribution • Biodistribution not limited to immune cells	[188, 193–196]
Extracellular vesicles (EVs)	Peptides, antibodies, and TLR agonists decorated targeting	Nucleic acids Tumor antigens Adjuvants Ligands Cas9 RNP	• Support T cell priming • Modular targeting	• Scalability and manufacturing limitations • Safety concerns	[264, 267, 268]
Mesoporous silica NPs	Surface particle functionalized targeting	Nucleic acids Protein antigens Adjuvants	• Co-delivery of adjuvant and antigen cargo • Chemically and thermostable • Surface functionalization possible	• Long-term fate dictated by silica pore chemistry • Needs optimization for stability	[194, 219, 220]
Gold (Au) NPs	Functionalized glycan or monosaccharide coating for endocytosis	Nucleic acids Antigens Adjuvants	• Tailorable physicochemical properties • Surface decoration possibility	• Accumulation and toxicity concerns • Size dictates NP biodistribution and DC maturation	[235–238]
Superparamagnetic iron-oxide NPs	Magnetic field manipulation and ligand-decorated targeting	Antigen, Metal ions STING agonists	• Labeling for DC tracking and migration • Magnetofection allows co-delivery of antigens and agonists • Enhanced anti-tumor response	• Limited clinical translation due to cytotoxicity	[226]
Zinc-based NPs	Functionalized with antigen moieties or tumor-derived peptides	Small molecules Neoantigens	• Possible for hybrid NP vaccines • High chelating ability and adjuvanticity • Efficient co-encapsulation of antigens	• Immunotoxicity • Risk of oxidative damage (ROS) upon localization • Off-target accumulation	[230, 231]

### Lipid nanoparticles (LNPs)

#### Empty LNPs

LNPs, comprising of lipophilic bilayer-forming clusters or spherical capsules, are well-studied nanoscale assemblies in intracellular drug delivery. The unique size, shape, and physicochemical properties of LNP favor efficient cellular uptake. Their similarities with the cell membrane composition and nanoscale sizes allow entry to cells via lipid exchange, membrane fusion, surface interactions, pinocytosis, and receptor-mediated endocytosis [203]. A typical LNP comprises an ionizable cationic lipid, sterol (cholesterol), polyethylene glycol (PEG)-lipid, and a helper lipid.

LNPs exhibit intrinsic immunomodulatory properties that can significantly affect targeted immune responses. For instance, ionizable lipids used in existing LNP formulations have a recognized *bona fide* adjuvant effect, independent of their payload [204, 205]. Recent LNP-mRNA formulations incorporate heterocyclic amine head groups into lipids, driving DC maturation via the STING signaling cascade rather than the TLR signaling. Furthermore, empty LNPs (eLNPs) elicit both innate and adaptive immune responses. This lipid-associated adjuvant effect synergizes with the encoded mRNA cargo for enhanced in vivo antitumor responses [206]. For example, eLNPs from various mRNA vaccines influence IL-6 production and follicular T helper (T_FH_) cell responses, coupled with the generation of antigen-specific germinal centre B cells and Ab-secreting plasma cells [205]. Thus, eLNPs can be suitable nanocarriers for targeted DC engineering for inducing an antitumor immune response.

Moreover, a study by Connors et al. explored how eLNPs activate innate immune responses by promoting the maturation of human CD11c^+^ monocyte-derived DC (MDDC) and further differentiation of T_FH_ cells [207]. Mechanistically, eLNPs were shown to induce phosphorylation of key signaling intermediates (TBK1 and IRF7) downstream of PRR activation. Prior mouse studies suggested that the immunogenicity of eLNPS was independent of canonical TLR/RIG-I, myeloid differentiation factor 88 (MYD88), and mitochondrial antiviral signaling (MAVS) pathways (44). eLNP-stimulated activation of STING/TBK1/IRF7 pathways in humans may indicate engagement of IFN-α/β receptors or possibly alternative cytosolic sensors [207]. Overall, using eLNPs may dictate the balance of vaccine adjuvanticity and innate immune activation, particularly for poorly immunogenic and immunosuppressive-prone therapeutic cancer immunotherapy and vaccines [208].

#### Cargo-loaded LNPs

While eLNPs are inherently immunogenic, the addition of an adjuvant greatly enhances immune response in patients [209, 210]. STING-mediated activation increases exogenous antigen cross-presentation in the cDC1 subset (31). Recently, Wang et al. (2024) reported lipid nanoparticle (LNP) delivery of a STING mimic (uniSTING) to DCs and metastatic tumor models, including triple-negative breast cancer, melanoma, and liver malignancies [211]. Encapsulation via LNPs enabled passive targeting and efficient intracellular delivery of uniSTING mRNA cargo. In DC-mediated therapies, the antigen escapes from lysosomal degradation favours MHC-I mediated cross-presentation and subsequent CD8⁺ T cell activation [212]. The uniSTING delivery into DC is also demonstrated to escape from lysosomal degradation, resulting in enhanced cross-presentation and infiltration of intratumoral granzyme B (GzmB^+^), intratumoral IFNγ^+^ cytotoxic CD8^+^ T cells.

Similarly, STING signaling in DCs induces transcriptional regulation of IFN regulatory factor 3 (IRF3) and NF-κB mediated secretion of pro-inflammatory cytokines, IFN-I, and chemokines [213, 214]. In DCs, the activation of the IRF3/IFN-I signaling cascade promotes antitumor activity, whereas the NF-κB/IL-6/STAT3 axis activation escalates tumor progression [215]. By imitating a cyclic guanosine monophosphate-adenosine monophosphate (cGAMP) -induced oligomerization, uniSTING selectively minimizes NF-κB proinflammatory activation while engaging IFN3/IFN-I signaling [216–218]. Interestingly, uniSTING can phosphorylate downstream signaling proteins TANK-binding kinase 1 TKB-1 and IRF3 to induce both IFN-β and interferon-stimulated gene (ISG) expression, in the absence of an endogenous STING signaling [211]. For maximizing the potential of DCs in cancer immunotherapy, more attention on nanocarrier formulations that activate cGAS/STING signaling axis in DC is critical.

### Silica nanoparticles

Similarly, an increase in cytosolic calcium ([Ca^2+^]_int_) promotes DC maturation. Ca^2+^ activates signaling pathways, including NFAT and NF-κB, promoting expression of co-stimulatory molecules in DCs [219, 220]. Nevertheless, a sustained increase in Ca^2+^ is required for full DC maturation [221]. Silica-layered calcium PEGylated Ca(OH)_2_/SiO_2_ core–shell NPs conjugated with anti-CD205 antibodies (AnCHNPs) are known to modulate DC function via artificial elevation of cytosolic [Ca]_int_. DCs sample their surroundings for antigens and undergo maturation, marked by upregulation of antigen-presenting molecules and co-stimulatory molecules such as CD80, CD86, and CCR7 [222, 223]. Notably, even in the presence of an immunosuppressive cytokine (IL-10), AnCHNPs enhanced DC maturation, as indicated by increased MHC-II expression and upregulation of CD205 in DCs.

### Metal nanoparticles

Metalloimmunotherapy leverages the natural immune response using metal and metal-derived NPs. The co-delivery of metal-derived NPs with STING agonists, such as cyclic di-adenosine monophosphate (cAMP) or cGAMP, manganese (Mn^2+^) amplifies the STING pathway and IFN-I production in DCs [224]. In clinical trials, STING activation for antitumor responses is derived mainly from CDA-based components [224]. Even in the absence of STING ligands, an assembly of cyclic dinucleotide (CDN)–Mn^2+^ (or CMP) can augment the phosphorylation of downstream targets (e.g., TBK-1, IRF) for DC activation [224]. To address the bioavailability and stability limitations of free Mn^2+^ and CDA from CMP admixture, CMPs can be developed as coordination polymers that co-deliver both components, using dose-sparing to minimize side effects.

#### Magnetic nanoparticles

RNA-loaded magnetic NPs with cationic liposomes also promote DC migration, enabling enhanced imaging of DCs in lymph nodes via non-invasive magnetic resonance imaging (MRI) [225]. In particular, superparamagnetic iron oxide (SPIO) NPs, although their cytotoxicity has posed limitations to clinical translation, are known to contribute to structural and functional features of NP delivery systems. Similarly, iron (III) oxide (Fe_3_O_4_)-Mn/Ca-based magnetic NPs effectively deliver OVA antigens to DC cytoplasm via magnetic field manipulation [226]. The active control of magnetic field increased particle contacts with DCs, thereby facilitating increased internalization of antigens. As reported, the addition of Mn^2+^ cargo stimulated DC maturation via the cGAS-STING signaling [226, 227] while [Ca^2+^]_int_ degradation modulated autophagy and enhanced Ag cross-presentation [228, 229].

#### Zinc nanoparticles

Efforts are made to engineer multifunctional SPIO with zinc oxide (ZnO) shell hybrid systems by conjugating them with carcinoembryonic antigen moieties or other tumor-targeting peptides for precise antigen delivery without additional transfection agents [230]. NPs with zinc (Zn^2+^) offer high co-encapsulation capacities for antigenic peptides, owing to the metal’s high chelating ability and interaction with the amino and phosphate groups of proteins [231]. Treatment using ZnO NPs highlighted ZnO-mediated inflammation, leading to DC-mediated maturation and subsequent release of proinflammatory cytokines (IL-6 and TNF-α) [232]. Comparably, incubation of DC membrane protein-derived zinc phosphate (ZnP) (LDC@ZnP) NPs increased similar inflammatory cytokines and upregulated CD11c^+^CD86^+^ BMDCs [9]. This immunostimulatory DC activation and maturation is attributed to the inherent adjuvant properties of ZnP [231]. Moreover, DC hybrid nanovaccine co-loaded with melanin and neoantigen ADP-dependent glucokinase (Adpgk) peptide suppressed murine colon adenocarcinoma (MC38) growth by eliciting the activation and proliferation of CD8^+^ CTLs and CD4^+^ helper T cells [233].

#### Gold nanoparticles

Compared to PEGylated particles, coating modifications with zwitterionic ligands of AuNPs and NCs demonstrated enhanced uptake and DC maturation, indicated by an increased CD80 expression. PEG-coated NCs showed reduced internalization, likely due to their interactions with cellular membranes and reduced recognition. Moreover, other applications, such as stabilization using Au shells, enabled straightforward ex vivo and in vivo tracking of DC migration to lymph nodes during PET/CT and CLI imaging [234]. Radionuclide-embedded AuNPs (RIe-AuNPs) exhibited improved intracellular stability without compromising phenotype markers (MHC I and II, CD86), cytokine secretion, and viability [234].

Considerations in NP size and surface modifications significantly impact the delivery of AuNPs to LNs through lymphatic vessels [235, 236]. Previous studies showed that smaller AuNCs (~ 2 nm) had higher internalization uptake than their > 12 nm AuNPs counterparts, likely due to enhanced diffusion and surface interactions [237, 238]. Interestingly, in terms of maturation, 2 nm AuNCs failed to induce maturation despite their superior uptake efficiency in DCs [238]. Contrary to these findings, larger AuNPs (~ 50 nm) demonstrated superior intake efficiency compared to 10 nm AuNPs, potentially due to their advantages with membrane dynamics [239]. The former induced IL-17 secretion, while 10 nm AuNPs suppressed HLA-DR. Thus, the paradox of efficient uptake not correlating with functional nature could be attributed to diverse intracellular signaling pathways triggered by NPs of varying sizes and surface chemistries. These findings challenge the notion that NP uptake may not follow a simple linear size-dependent trend and can be modulated by additional factors such as shape and functionalization.

Overall, inorganic nanoparticles provide significant advantages in DC targeting. Despite this, compared to other nanocarriers (Table 4), their higher toxicity and immunogenicity require complex formulations to ensure biocompatibility and targeted delivery.

### Nanoparticles and DC receptor targeting

#### CD169 (Siglec-1)

Several receptors can be targeted or attached to DCs for potential cell-specific targeting. PRRs on DCs hold promise in targeted immune activation. The sialic acid binding Ig-like lectin 1 (Siglec-1 or CD169) is a receptor expressed on splenic macrophages known to mediate cell-to-cell interactions. CD169^+^ macrophages assist in pathogen capture and antigen transfer to DCs to initiate an effective T cell response. Further, the IFN-1 produced by CD169^+^ macrophages and pDCs enhances DC maturation and cross-priming of CD8^+^ T cells. Single-cell RNA sequencing revealed that human CD123^+^ AXL^+^ DCs expressed CD169 receptors, which assisted them in capturing HIV particles upon infection [207]. The addition of its ligand, ganglioside GM3, to liposomes promotes antitumor CD8^+^ T cell responses [240].

#### CD40

Similarly, the conjugation with anti-CD40 antibodies directs NPs to CD40^+^ DCs. An interaction with its ligand (CD40L) induces DC maturation and CD4^+^ T_H_ cell-dependent CD8^+^ T cell priming [209]. Encapsulation with adjuvants (TLR1/2 ligand, Pam3CSK4; poly[I:C], polyinosinic:polycytidylic acid) and antigen (HPV-E7) into biodegradable poly(lactic-co-glycolic acid) NPs (PGLA_αCD40-mAb_) enabled selective DC delivery and effective B16-OVA melanoma tumor killing, with HPV-E7-specific CD8^+^ T cell immune response [241]. However, without TLR ligands, PGLA_αCD40-mAb_-Ag generated poorly matured DCs. Despite the promising potential of targeting CD40, αCD40-mAb-related toxicity has been reported at higher doses, suggesting localized delivery of a slow-release formula in CD40-based targeting [242].

#### Fc receptors (FcRs)

Fc receptors (FcRs) in DCs have been utilized for targeted vaccine delivery. NPs can target Fc receptors via Fc fragments or full antibodies, with most studies focusing on Fcγ receptors [243]. For example, IgG-coated liposomal vaccines with OVA, in contrast to non-coated liposome constructs, are known to suppress the development of OVA-expressing lymphoma in mice [244]. Liposomal L-rhamnose-conjugated vaccines with Pam_3_CysSK_4_-MUC1/OVA showed promising results for better Ag presentation to MHC classes [245]. Fc receptors on DCs recognize the Fc region of bound anti-rhamnose Ab, facilitating DC maturation after internalization of the cancer vaccine Pam_3_CysSK_4_-DBCO with tumor-associated carbohydrate antigen (glycoprotein MUC1-Tn). Moreover, Fc conjugation to liposomal vaccines can enhance both humoral and cellular immune responses when tumor peptides are included in the NP formulation [246].

Whereas FcRs are expressed on diverse cell types, DC-targeted vaccines have focused on specific DC subsets, with the antigen delivered using mAbs to receptors with restricted expression to the subset of particular interest. Among widely studied examples are C-type lectin receptors (CLRs) and X-C motif chemokine receptor 1 (XCR1).

#### DC205 and DCIR2

DEC-205 (CD205), a receptor for type B oligonucleotides, is expressed in cDC1s and other cell types (cDC2s and moDCs) [247]. PLGA NPs conjugated with monoclonal αDEC-205 antibodies enhanced melanoma-associated Ag internalization and cross-presentation in DCs, leading to superior activation of CD8^+^ T cells compared to non-targeted NPs [248]. Also, targeting DEC-205 led to preferential cross-presentation in T cells. For antigen presentation to CD8^+^ T cells, DEC-205 is targeted to CD8^+^ DCs; likewise, focusing on DC inhibitory receptor-2 (DCIR2), a highly expressed CLR receptor in cDC2, initiates a specialized cross-presentation capacity to CD4^+^ T cells [249]. DCIR2 is expressed on poorly cross-presenting CD8^−^ DCs, while DEC-205 is highly expressed on CD8^+^ DCs [250]. However, careful considerations must be given to the receptor of choice, as DEC-205 targeting has been shown to induce DC tolerogenicity depending on the formulation.

#### Clec9A

Targeting Clec9A, another CLR specifically expressed on cDC1s, has shown promise in eliciting strong antitumor responses. The BDCA3^+^ DC subset in humans, which corresponds to mouse CD8^+^ DCs, expresses Clec9A and may be a promising target for inducing cellular immune responses [251]. This is due to the potent cross-presentation capability of cDC1s, which are critical for initiating cytotoxic T cell responses [252]. Previous reports discuss NP combinatorial targeting of BDCA3^+^ and monocyte-derived DC-SIGN^+^ DCs eliciting IL-15-dependent T cell activation compared to targeting either subset alone [253]. Also, targeting Clec9A with NPs loaded with TAAs (e.g., Trp2 and gp100) induced NKT and CD8^+^ T cell expansion in healthy donors and melanoma patients [254].

#### DC-SIGN

Targeting DC-specific ICAM3-grabbing non-integrin (DC-SIGN; CD209) is a type II transmembrane CLR expressed in macrophages, moDCs, CD14^+^ dermal DCs, and DCs at mucosal sites. Antigens presented to this receptor are efficiently processed for MHC I /II cross-presentation [255]. In preclinical studies, targeting DC-SIGN with lipo-Lewis Y (Le^Y^), formulated with its natural glycan ligand Le^Y^, enhanced in vitro and ex vivo CD8^+^ T cell responses [256]. It was found that incorporation of Le^Y^ to liposomes for directed delivery augments gp100 presentation by moDCs, dermal DCs, and Langerhans cells, suggesting potential usage in boosting antitumor responses [256]. Yet, the phase I clinical trial of a multicomponent DC-targeted liposomal vaccine (Lipovaxin-MM carrying gp100 and MART-1) did not produce significant antitumor activity (i.e., no detected immunogenicity despite being well tolerated) [257].

In the context of nanovaccines, a combined approach using large-pore silica NPs (Si9GM) enabled DC maturation, cross-presentation, and potent antitumor immunity [209]. Si9GM, derived from BMDCs, specifically targets cDC1s via the Clec9A receptor. Upon entry into DCs, this nanovaccine delivers the OVA_257-264_ antigen to CD8^+^ T cells and stimulates the STING pathway with the 2′3′-cGAMP agonist inside the Si9GM pore. In addition, Si9GM triggers the production of IFN-I and proinflammatory molecules from the activation of several key proteins, including TBK1, IRF3, and NF-κB. In contrast with free STING agonists, Si9GM freely escapes the lysosome, allowing component delivery into the cytosol for immune activation.

While the conjugation and decoration of DC ligands in active targeting seem like a straightforward process, the stability of the resulting LNP may be compromised. That is why both targeting moieties in functionalization and physicochemical properties in formulation should be considered. Interestingly, organic nanoparticles assist in overcoming these limitations in DC-targeted delivery applications.

### Extracellular vesicles in DC targeting

Extracellular vesicles (EVs) represent a highly diverse group of membrane-bound particles that serve crucial roles in intercellular communication, immunity, and disease pathology. They are classified into exosomes, microvesicles (MVs), and apoptotic bodies, based on their size, biogenesis, and molecular composition. Recently, EVs have been expanded to include large oncosomes, migrasomes, ectosomes, exomeres, supermeres, and membrane particles [258, 259].

Exosomes are 30 to 150 nm sized multivesicular bodies (MVBs) that are generated through the inward budding of endosomal membranes [258]. Their ability to fuse with the plasma membrane, releasing their contents into the extracellular space, makes them an attractive candidate for drug delivery applications. The exosomes are enriched with tetraspanins, integrins, and other cell-specific markers that reflect their origin [259].

Cells can communicate with each other via exosomal cytokines and miRNAs, and they have been reported to work in tandem to regulate dendritic cell (DC) maturation and immune responses. For instance, exosomal miRNAs, such as miR-155 and miR-146a, modulate key signaling pathways that regulate DC differentiation, antigen presentation, and cytokine production [260]. In cancer, tumor-derived exosomes (TEx) have been demonstrated to internalize or fuse with DCs, delivering tumor-associated antigens in the form of membrane-bound proteins and MHC class I/II–peptide complexes [260].

Microvesicles, on the other hand, are larger vesicles, usually between 100 and 1000 nm, formed through outward budding from the plasma membrane and have been reported to contribute to immune activation by transporting inflammatory cytokines [261], chemokines, and danger-associated molecular patterns (DAMPs) [262]. Apoptotic bodies, often exceeding 1000 nm, arise during programmed cell death and contain cellular organelles, fragmented nuclear material, and various cytoplasmic components^6^. Apoptotic bodies were reported to carry CX3CL1 (fractalkine) and CCL2, which enhance monocyte and macrophage recruitment to sites of tissue damage, initiating a local immune response.

The unique ability of exosomes to carry diverse cargoes, surface functionalization, lower immunogenicity, potential to cross the blood–brain barrier, higher biocompatibility, and site-specific delivery hold great promise for targeting DCs in cancer immunotherapy [263].

#### Exosomes and exosomal engineering for DC targeting

Exosomes are an attractive target for drug delivery to DCs due to their nanosize, ability to pass through tight junctions, and their dynamic interactions with DCs to modulate immune response [263]. Advancements in exosome engineering have further increased the appeal of exosomes as DC-modifying agents.

**Fig. 6:**
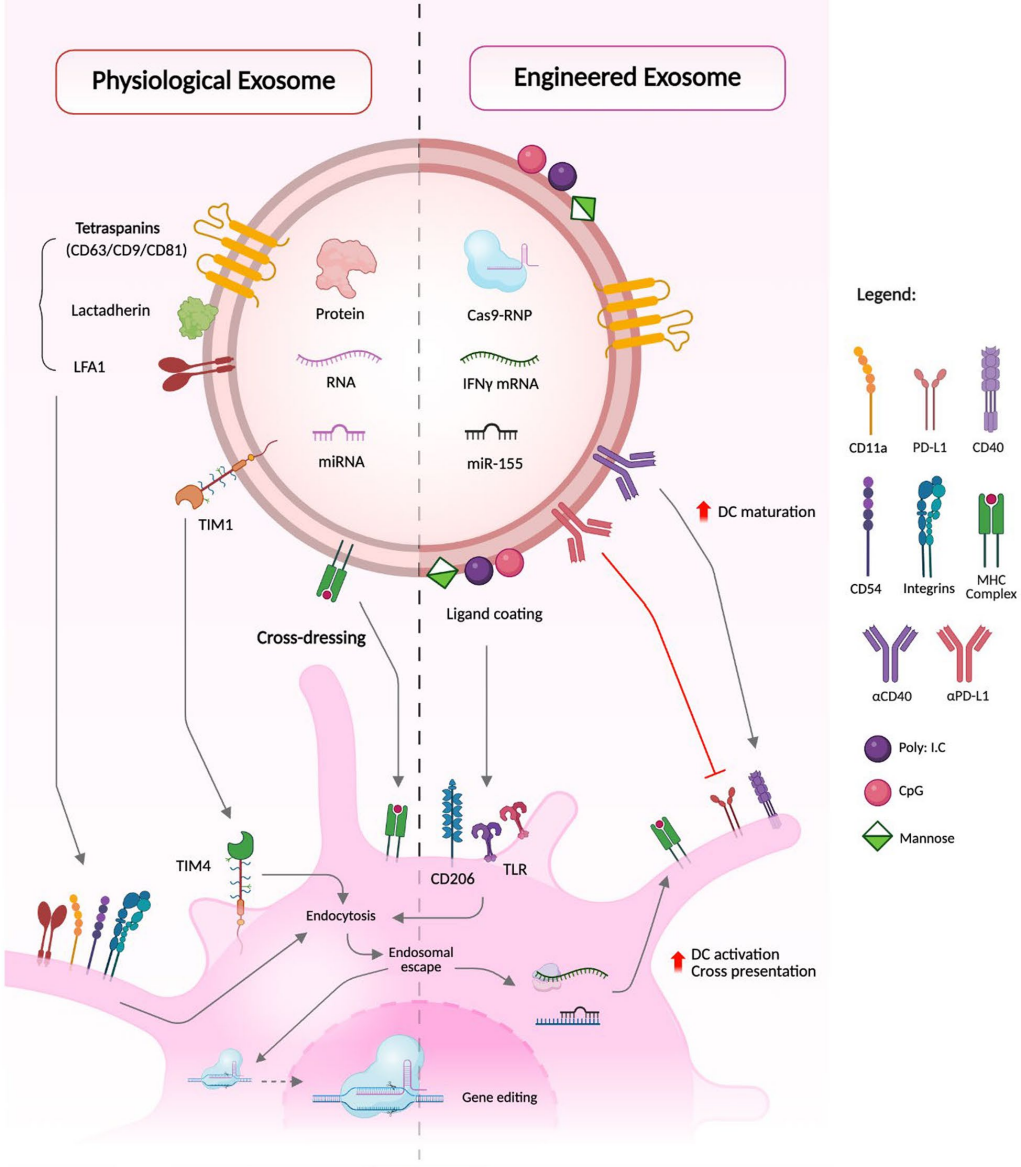
Functional mechanisms of physiological and engineered exosomes in DC modulation. Physiological exosomes carry biomolecules such as proteins, RNAs, miRNAs, and MHC complexes, and interact with DCs via receptors like TIM1 and TIM4, leading to endocytosis or antigen cross-dressing through the transfer of peptide–MHC complexes. Engineered exosomes are modified with functional cargos (Cas9–RNP, IFN mRNA, miR-155) and targeting ligands, antibody fragments (anti-CD40, anti–PD-L1), or immunostimulatory motifs (integrins, mannose, Poly I:C, CpG). These modifications enhance DC uptake, endosomal escape, and immune activation, supporting applications in immunotherapy and gene delivery. Figure illustrated with BioRender

Surface modifications of exosome improve exosome-cell interactions, ensuring selective uptake by DCs **(**Fig. 6**)**. Exosomes could also be coated with peptides, antibodies, and TLR agonists to improve immune function and activation [264]. For example, Poly(I:C)-coated exosomes activate TLR3 pathways and have been reported to amplify innate immune responses [265], while CpG-modified vesicles were documented to engage TLR9, promoting antigen presentation [266]. Mannose-modified exosomes bind mannose receptors on DCs, improving antigen processing efficiency, whereas glycan-modified exosomes engage C-type lectin receptors, optimizing cross-presentation pathways [267, 268]. Antibody-conjugated exosomes, such as anti-CD40-engineered vesicles, enhance DC maturation and immune priming. On the other hand, anti-PD-L1-modified exosomes help reverse tumor-induced immunosuppression [269]. Exosomes can also be loaded with different cargoes, modulating the immune cells they target. For instance, miRNA-loaded exosomes, such as those containing miR-155, regulate DC maturation and antigen presentation, improving immune responses. Furthermore, mRNA-loaded exosomes have also been reported to encode cytokines like IFN-γ, which enhances antiviral pathways and immune activation [270, 271]. These enhancements enhance DC functions and are particularly advantageous for therapeutic intervention.

### NP-mediated delivery of CRISPR/Cas9 system to DC

The advent of CRISPR/Cas9-assisted precise editing of genes has gained traction, and its applications are linked to cancer therapeutics and immune regulations [272]. Yet, most immune cell reprogramming efforts focused only on T cells, with limited work targeting DCs [273]. Despite significant promises of CRISPR-based treatments, delivery challenges hinder clinical applications. The high nuclease degradation capacity of DCs during phagocytosis warrants efficient delivery approaches for Cas9 mRNA and guide RNA for successful gene editing [274]. NPs with favorable biocompatibility, encapsulation capacity, and adjustable surface features offer promising platforms for delivering gene editing tools to DCs.

Zhang et al*.* designed a lipid-assisted nanomedicine comprised of Cas9 mRNA and CD40-targeting gRNA, encapsulated within poly(ethylene glycol)-*block*-poly(lactide-*co*-glycolide) (PEG-*b*-PLGA)-based NPs. This formulation was enhanced with a cationic lipid BHEM-Chol, resulting in cationic lipid-assisted NPs or CLAN. Zhang et al*.* investigated the potential of CLAN in DC reprogramming and transplantation tolerance in mice [156, 275]. Treatment with CLAN_mCas9/gCD40_ significantly reduced the expression of CD40, CD80, and CD86, contributing to T cell anergy and reducing graft rejection in mice. The CLAN_mCas9/gCD40_ formulation could be adopted for targeted engineering of DCs to boost their antitumor response [276].

BAMEA-O16B is another nanoformulation that delivers both Cas9 mRNA and sgRNA to tumors, human embryonic kidney cell lines, and mouse hepatocytes in vivo [277]. Mao et al. developed BAMEA-O16B lipid-assisted NPs (BLANs) for efficient RNA cargo encapsulation [116]. BLANs with lower cholesterol exhibited efficient mRNA encapsulation rate, higher zeta potential, and larger particle sizes. Further, the authors developed BLANs with Cas9 mRNA and sgRNA targeting PD-L1 in cDC1 (BLAN_mCas9/gPD-L1_). The low cholesterol BLAN_mCas9/gPD-L1_ formulation showed enhanced cellular uptake with efficient release of gene editing components, leading to a successful PD-L1 knockout in cDC1s. The selective PD-L1 targeting in cDC1 resulted in tumor growth suppression via T cell promotion and a heightened cDC1 activation [278].

Exosomes serve as efficient vehicles for CRISPR-Cas9 delivery, with encapsulation methods falling into three primary categories: physical, chemical, and biological approaches [279]. Physical loading methods, such as electroporation, offer high encapsulation efficiency but risk membrane disruption [280]. Saponin treatment stabilizes Cas9 RNP complexes with moderate efficiency, requiring toxicity management, while ultrasound treatment enhances membrane permeability but demonstrates lower efficiency [281, 282]. Chemical methods**,** including cationic lipid conjugation, improve protein stability and intracellular transport, whereas the Exo-Fect reagent streamlines encapsulation but shows limited efficiency [283, 284]. While physical and chemical techniques offer scalability and simplified handling, concerns related to exosome integrity, cytotoxicity, and reproducibility must be addressed for clinical applications [285].

Biological engineering strategies provide precise cargo loading by leveraging molecular interactions. Affinity-based systems, such as GFP/GFP-nanobody fusion, enhance RNP encapsulation specificity [286], while myristoylation achieves 0.7% loading efficiency but suffers from stability issues [287]. Heterodimerization strategies, including Fc/Protein A interactions and light-induced dimerization (Cryptochrome 2), offer modular control over exosome content [288]. Aptamer-based loading (e.g., MS2/MS2 coat protein, Com/Com-aptamer) improves cargo stabilization [289]. Chemical induction, such as AP21967-triggered dimerization using FKBP12/FRB, enables 3.5–7.9 RNP molecules per exosome, significantly enhancing gene editing precision [290]. Though biologically engineered approaches offer superior specificity, their reliance on cellular modifications introduces regulatory complexities that require further refinement for therapeutic translation.

Delivery strategies in the nanoscale assembly have advanced to focusing on educating APCs, particularly innate sentinels such as DCs, rather than targeting cancer cells alone. Beyond prolonged circulation, effective cell targeting, and precise delivery of antigen and adjuvant cargoes, current NPs encapsulate gene editing tools. Cell-based, organic, and inorganic NPs each offer unique advantages in native cell interaction (biocompatibility), biodistribution, and surface functionalization [291, 292]. Yet, despite these opportunities, NPs still face challenges involving inherent toxicity, induced immunogenicity, and limited scalability. Moving forward, optimizing features such as surface functionalization for receptor targeting, stealth, size, and endosomal escape is critical for leveraging DC-targeted NPs in clinical trials. Such integration of surface modifications and optimized Cas9 cargo loading strategies in NP formulations provides a highly suitable platform for DC-targeted gene editing and immune therapy. Efforts for future research must focus on enhancing ligand specificity, optimizing biomanufacturing scalability, and refining endosomal escape mechanisms to ensure precise cargo delivery genome editing with minimal off-target effects. These advancements will further solidify NP-cargo delivery and CRISPR-Cas9 gene editing efforts in DCs as next-generation therapeutic tools in cancer immunotherapy, personalized vaccine strategies, and immune modulation.

## Future perspectives

DC-based cancer immunotherapy has advanced over the past years, and strategies to improve its functions are continuously being optimized. While employing DCs for preventing cancer, a crucial factor to consider is the constant immunosuppressive state generated by the TME, which weakens DC function and impairs T cell activation (Fig. 2) [54]. The tumor generates metabolites, cytokines, and apoptotic bodies in TME that suppress the cross-presentation and cytokine signaling in DC. Further, TME-induced upregulation of IDO-1 activates kynurenine-mediated immunosuppressive events in DC that dampen T cell activation significantly. In addition, the immune inhibitory PD-L1/PD-1 interaction between DC and T cells drives T cell exhaustion [54]. Thus, it is crucial to understand the factors that inhibit the immune responses in DCs and develop approaches to mitigate them to completely harness the potential of DCs in cancer immunotherapy. Identifying appropriate DC subsets and gene targets through genome editing tools allows permanent DC engineering, boosting their T cell activation and antitumor potential. These strategies make DC a suitable target for cell therapy and immunotherapy for cancer.

### Screening immunostimulatory targets

Recent developments in CRISPR/Cas9-enabled genome editing strategies facilitate precise DC engineering and present diverse opportunities for overcoming tumor-induced immunosuppression. While CRISPR/Cas9-mediated DC editing is still in its initial stages, combining the potential of CRISPR/Cas9 genome screening with in-depth knowledge of DC biology is critical for identifying viable immunostimulatory targets. Although human moDCs and murine BMDCs are reported for CRISPR screening [139, 153, 154], identifying the DC subset with high cross-presentation capacity and the methods to isolate and expand them for CRISPR/Cas9 genome screening still need much attention.

### Enhance DC-T interaction

CRISPR/Cas9-directed insertion of TLR agonists, tumor neoantigens, or co-stimulatory molecules to induce endogenous DC maturation ensures promising antitumor immunogenic activity [135, 142]. Cancer cells engage in several immunosuppressive interactions with DCs to disable their T cell-activating potential [78]. Hence, knocking out immunosuppressive receptors using CRISPR/Cas9 targeting in DCs is a promising approach. For instance, CRISPR-mediated silencing of immune checkpoint mediators such as SOCS1 and PD-L1 in DCs has heightened T cell activation responses in preclinical models [134]. In addition, combining modifications in DCs that enhance their maturation, activation, and migration with silencing immunosuppressive signals may sustain potent antitumor T cell responses.

### Manipulate DC-TME interaction

A major function of DCs in immune response is phagocytosis. Tumor cells evade phagocytosis by upregulating CD47 expression [129]. This “Don’t eat me!” signal prevents their engulfment by DCs to suppress immune defenses. Several malignancies positively correlate with CD47 expression and CD47-signal regulatory protein-α (SIRP-α) interaction to evade phagocytic activity [129]. Efforts to generate antibodies to inhibit CD47 and SIRP-α were futile due to their severe toxicities to normal cells [130]. CRISPR/Cas9-mediated knock-out of SIRP-α in DCs presents a promising immunotherapeutic target to avoid the ‘Don’t eat me!’ signal from tumors and enhance their phagocytic potential. Another strategy involves boosting neoantigen generation in the TME. The DNA mismatch repair gene (MLH1) is critical for correcting errors created during DNA replication, ensuring genome stability and cell viability. MLH1 expression is positively correlated with many cancer types, for example, colorectal adenocarcinoma [293, 294]. In such cancers, CRISPR/Cas9-guided knock-out of the DNA mismatch repair gene (MLH1) in tumor cells increases their mutational burdens. This generates neoantigens suitable for DC uptake and cross-presentation to CD8^+^ T cells [293].

### DC subset identification

In parallel, focusing on specific DC subsets with potent T cell activation ability is another approach to improve cancer immunotherapy. More research harnessing the potential of different DC subsets (cDC1, cDC2, and pDCs) in inducing antitumor response is critical for inducing an intense T cell response. Methods to isolate and expand the DC subsets with cross-presentation ability are needed for effective tumor suppression. DC-based cancer therapies initially focused on moDCs due to their relative accessibility [10]. Although efficient in in vitro cross-presentation, moDCs were not potent CD8^+^ T cell activators in vivo because of their limited migratory capacity [10]. Targeting multiple gene editing in moDCs that results in enhanced TAA and CCR7 expression with simultaneous suppression of E-Cadherin could be an effective resolution to use moDCs for cancer therapy [145]. Autologous cDC2 and pDCs are considered a feasible alternative approach in DC therapy. However, to establish them in clinical studies, a thorough understanding of their anti-cancer mode of action and flexibility for engineering is required.

cDC1 is the most favourable DC subset in cancer therapy because of its unique potential to activate both CD8^+^ and CD4^+^ T cells [295]. They are also the major producers of IL-12, which is critical for anti-cancer immunity. Isolating the cross-presenting cDC1 population presents considerable challenges because of the difficulty in achieving enrichment in cultures from both humans and mice [296]. This is mainly because of the insufficient expression of crucial markers that are essential for initiating an effective antitumor response.

### Somatic cell reprogramming to cDC1

Somatic cell reprogramming to express key developmental factors of cDC1 is a promising alternative strategy for generating immune-responsive cDC1s. A major milestone in this area was achieved by successfully reprogramming dermal fibroblasts to cDC1 [297]. The murine embryonic fibroblasts and human adult dermal fibroblasts were engineered to cDC1-like cells by introducing LVs encoding for cDC1 developmental factors such as BATF3, IRF8, and PU.1 [298]. scRNA seq analysis of the murine reprogrammed MHC II^+^/I^+^ XCR1^+^ induced DCs clustered them closely with primary splenic cDC1s and expressed maturation markers CD40, CD86, and IL-12 upon TLR stimulation [10, 297, 298]. Like conventional cDC1s, the generated iDCs exhibited the capacity to capture, process, and present antigens to activate CD8^+^ T cells [297]. However, the human adult dermal fibroblasts reprogrammed into CD141^+^ CLEC9A^+^ iDCs demonstrated low efficiency in HLA-DR expression [10]. The limited reprogramming efficiency of LV-mediated gene editing is a major limitation that must be addressed for the wide-scale applicability of this reprogramming technique.

### Nanocarriers in DC cancer immunotherapy

Harnessing the potential of DCs using NPs for controlled immune responses holds great promise for elevating the current platform for antitumor responses. However, many areas of DC targeting nanodrug delivery are regarded as unclear owing to the complexity of NP formulation, toxicity, and challenges in the interactions of nano-bio responses, hampering translational success. Some of the key hurdles that require immediate attention are challenges in real-time tracking, poor encapsulation, antigen delivery, and immune activation. The functionalization and combinatorial targeting of different DC-specific receptors, such as DC-SIGN, DEC-205, and Clec9a, hold significant promise in improving current treatments. The targeting receptor, dose, and NP formulation are critical factors that dictate its immunogenicity or tolerogenicity.

Such successes of LNP formulations enabled the clinical application of mRNA payload, particularly the development of COVID-19 vaccines. As such, LNPs can be suitable nanocarrier candidates for DC-targeted drug delivery applications for cancer immunotherapy. While the first FDA-approved LNP for RNA interference (RNAi) achieved therapeutic relevance, its application is limited to hepatocytes and related liver diseases. The need for developing delivery systems that target other organs after systemic administration. Thus, the first step in targeting SLOs is to avoid unnecessary uptake by the liver. Fine-tuning of lipid ratios (helper lipid dioleoyl phosphatidylethanolamine [DOPE], pH-sensitive lipid 1,2-dioleoyl-3-dimethylammonium propane [DODAP], DMG-PEG2k, and cholesterol) allows successful cargo delivery and protein expression by mRNA-loaded LNPs to splenocytes such as CD11c^+^ DCs after intravenous administration [299].

Phosphatidylserine (PS), a natural phagocytic recognition signal, improves active phagocytic targeting in SLOs. Inclusion of PS in LNPs (PS-LNPs) imparts active SLO targeting strategies compared to LNPs with lipid 1,2-dioleoyl-sn-glycero-3-phosphate (18PA-LNPs) and DLin-MC3-DMA [300]. In turn, the composition of corona proteins is dictated by the overall charge of the resulting LNP from the altered lipid composition, specifically the biomimetic functional group phosphoserine. While increasing the ratio of anionic lipid components improves splenic biodistribution, the caveat lies on the reduced mRNA expression in the spleen. Thus, advancing the adoption of LNPs for cell-specific SLO-directed targeting in cancer immunotherapy requires strategies that design LNPs with biomimetic properties, moving beyond just the idea of charge-dependent organ-specific localization.

Whereas ionizable lipids found in eLNPs could be suitable choices for therapeutics due to their ability to promote DC maturation and activation, their toxicity to DC is a key aspect that warrants utmost consideration during the novel NP formulation. The cytotoxic activity of cationic liposomes by forming transient holes in the cell membrane raises a challenge for DC-targeted applications [301]. In this case, future formulations should focus on the choice of ionizable lipids and corona decoration to reduce both nonspecific delivery and cytotoxicity.

In genome editing, LNPs loaded with Cas9 protein are increasingly reported for tumor reprogramming [302–304]. The large size and cationic nature of Cas9 pose a challenge for efficient encapsulation for targeted delivery to DC. However, Cas9/sgRNA RNP encapsulation overcomes this hurdle due to the presence of highly anionic sgRNA [305]. Hence, NP formulations aimed at delivering gene editing tools to DC need careful knowledge of electrostatic interactions between biomolecules and nanocarriers.

DC engineering to boost the efficacy of cancer immunotherapies is an emerging area of global interest. More studies need to be focused on developing nanocarriers that can efficiently carry Cas9-guide RNA RNP complexes into DCs without affecting DC viability or differentiation potential. Furthermore, identifying novel surface receptors on different DC subsets using CRISPR/Cas9 technology will assist in the formulation of nanocarriers suitable for DC receptor-ligand-mediated cargo targeting.

### Clinical translation

Ex vivo DC therapy, in which DCs are isolated from a patient's blood, manipulated with whole tumor cells or purified/recombinant tumor antigen peptides ex vivo, and then reinfused into the patient to activate an antitumor immune response, has garnered considerable appreciation [140]. Comprehensive reports on the clinical trials involving DC-based vaccines for cancer immunotherapy targeting multiple cancers have been reviewed elsewhere [306–311].

Several challenges, including immunosuppressive TME, DC subsets with sub-optimal cross-presentation ability, migratory ability of DC, TAA selection, and limited clinical translation due to safety concerns, restrict their wide-scale applicability [140]. The major considerations to make while transitioning antitumor DC therapeutics to clinical applications include enhancing the abundance and the immunogenic functions of DCs in the tumor and TdLNs [311].

One of the major obstacles in the clinical translation of DC therapy is the source of DCs. moDCs are routinely employed for developing DC vaccines due to their accessibility. The clinical trials using first-generation DC vaccines, such as the FDA-approved Provenge in 2010, use GM-CSF programmed moDCs pulsed with prostate tumor antigen. The limited efficacy and patient survival response of Provenge is due to compromised LN migration and antigen presentation capacity of moDCs [10, 311]. APCEDEN®, an autologous DC-based vaccine approved by the Indian FDA in 2017, uses moDCs pulsed with whole tumor lysate from the patients for the management of prostate cancer, ovarian cancer, non-small cell lung carcinoma, and colorectal cancer [312]. A clinical study conducted by Kumar et al. reported tumor regression in patients (3.0 × 2.5 cm to 2.1 × 1.6 cm) and decreased neutrophil to lymphocyte ratio (NLR) after 14 months of APCEDEN® therapy in parallel with Gefitinib administration. The restricted overall success rate of APCEDEN® due to the TME, prolonged response time, and cost limit their clinical application [312].

CRISPR-mediated DC engineering is a promising approach to ensure the successful application of DC therapy in cancer. CRISPR-based knock-in of migratory marker CCR7, co-stimulatory molecule CD70, and WDFY4 to enhance antigen cross-presentation, while knock-out of IL-10, IDO1, and adhesion molecules offers an attractive choice to enhance the efficacy of moDCs’ function in DC therapy. Another strategy is to enhance DC-MHC cross-dressing in moDCs with tumor antigen using a lentivirus-encoded chimeric receptor named extracellular vesicle internalizing receptor, EVIR [313]. Hence, using innovative CRISPR-based engineering strategies, moDCs used in DC therapy can be transformed into an efficient cross-presenting DC subset-like phenotype. Such strategies also offer promising outcomes in combination therapy in clinical settings.

Identifying DC subsets that exhibit elevated antigen cross-presentation and migratory potential is a critical factor in deciding the success rate of clinical trials using DC therapeutics. The scarcity of immunoregulatory DCs in the tumor restricts efficient CTL responses in patients. Clinical studies using the cDC1 population have gained considerable appreciation in recent years due to their superior CTL activation potential. Approaches to enhance the abundance of cDC1 population in the tumor and TdLNs are important. In situ vaccination with FLT3L and Poly (I:C) combination in indolent non-Hodgkin’s lymphoma patients showed considerable expansion of intratumoral DCs and high antitumor immune responses [306, 311]. The abundance of DCs in the tumor upon systemic administration of FLT3L has also been reported in recent clinical studies. Thus, strategies enhancing the abundance of cDC1 in TME offer better prognosis and patient survival by activating a rapid CTL immune response [314].

CD40 agonism in DC has been well established in the clinical translation of DC therapy. Second-generation Fc-engineered variants for CD40 agonism are already in clinical trials for bladder cancer (NCT05126472), gliomas (NCT04547777), and solid cancers (NCT04059588) [315]. Approaches combining CD40 agonism with CRISPR-engineered DCs, such as inhibiting immunosuppressive factors and expressing maturation markers, can offer promising anti-cancer therapeutic performance [316]. Spatial investigation of DCs is critical to understanding the therapeutic efficacy of DCs in the tumor site and TdLNs.

The clinical efficacy of DC-based cancer immunotherapies is enhanced by the use of targeted nanoparticles in the formulation. The RNA-lipoplex (BioNTech) mRNA NPs, either alone or with checkpoint inhibitors such as atezolizumab (BNT122; NCT05968326 and NCT03289962), or cemiplimab (BNT111; NCT04526899), demonstrated durable T cell responses induction upon intravenous delivery of personalized neoantigens [317, 318]. For subcutaneous delivery, amphiphile platforms (NCT04853017, NCT05726864) carrying antigenic peptides (albumin with KRAS/NRAS) and adjuvants to the lymph nodes offer designs for APC activation patrolling the area [319]. The NPs also maximize antitumor responses after intratumoral activation. For instance, in melanoma skin cancer, the activation of pDCs using virus-like particles (VLPs) containing a TLR9 agonist (vidutolimod/CMP-001, NCT04401995) in combination with anti-PD-1 nivolumab activated effector T cells [320]. Similarly, BO-112 is an intratumorally administered poly(I:C)-polyethylenimine formulated cationic nanoplex that activates TLR3/MDA5 axis [320]. In 2025, Phase 2 trials of BO-112 (NCT04570332) and pembrolizumab reported promising results in patients with progressive PD-1-resistant malignant melanoma [321].

In summary, our review points out that the current limitations faced by DC vaccines can be overcome with novel engineering strategies and targeted nanoparticle formulations. While several cancer immunotherapy clinical trials utilizing nanoplatforms target immune cells in general, the strategies mentioned in this review exemplify systemic DC targeting. Collectively, these platforms show diverse strategies for drug delivery and combinatorial immunotherapies. The future of NP formulation and clinical development emphasizes the need for highly versatile, targeted DC vaccine monotherapies with minimal drug toxicity.

## Conclusion

In this review, we discuss how the TME exerts immunosuppressive effects on DC during tumor progression and the precise genome editing strategies aimed at overcoming these effects in DC (Table 5). Nanoscale delivery strategies have advanced beyond targeting tumors to focus on educating APCs such as DCs. Gaining an in-depth understanding of the TME and its effects facilitates the tailored engineering of nanocarriers that enable efficient uptake and delivery of gene editing tools, such as Cas9, gRNAs, mRNAs, TLR agonists, and maturation inducers in DC in vivo. This significantly reduces the complications associated with the ex vivo modification of DCs in therapeutic applications. Smart delivery materials capable of carrying multiple cargoes with a TME-responsive release strategy represent a critical milestone achieved in DC-based cancer therapy. Furthermore, engineering nanocarriers that target DC subsets with enhanced cross-presentation ability is an important area that requires more attention. In conclusion, combining multiple treatment modalities, such as immune checkpoint inhibitors with genome-engineering in DC, offers promising synergistic outcomes in tumor suppression. However, continuous preclinical and clinical efforts must address the safety of these innovative tactics. Specifically, the gene editing in DC must be carefully validated for its off-target effects and unintended impacts on DC function. With careful considerations in translation strategies and nanoparticle architecture, integrating the perspectives of DC biology, functional genomics, and immunology with nanomedicine presents a promising therapeutic platform that ensures successful cancer treatment. Ultimately, combining DC-targeting strategies with specific genome editing approaches as precision nanomedicine offers significant potential for next-generation cancer immunotherapy.

**Table 5:** Overview of TME-induced dysfunctions in DCs and potential engineering strategies with efficient nanoparticle delivery methods

TME induced dysfunctions	Events	Engineering strategies	Promising targeted NP designs	References
Metabolic reprograming	Hypoxia-induced lactate uptake	• Knockout of CD36	• Biomimetic, amphiphilic macromolecules for CD36 binding • CD36 silencing by octaarginine (R8)-modified lipid envelope	[73, 345, 346]
	Enhanced HIF1α activity	• Knockout of *HIF1α*	• *HIF1α* siRNA lipid–calcium–phosphate (LCP) silencing	[69, 74, 347,348]
	Higher Fatty acid metabolism	• Knockout of carnitine palmitoyl-transferase I (*Cpt1*)	• NPs loaded with *c-Myc* siRNA-induced modulation of *Cpt1a*	[349, 325]
	Higher Lipid accumulation	• Knockout of *LxRα*	• Exosomal CRISPR/Cas9 RNP-loaded editing	[81, 98,^100^, ^350^]
	Higher ER stress	• Knockout of *XBP1*	• *XBP1* shRNA LNP silencing	[81, 81, 97, 351]
	Impaired autophagy pathway	• Enhance Atg5 activation	• *Atg5-*encoding mRNA LNPs	[73, 352]
	Higher arginase-1 activity	• Knockout of *Arg1*	• *Arg1* si/shRNA LNP	[353, 354–356]
	Higher adenosine activity	• Knockout or loss of function of *A2A* and *A2B* receptors	• NP co-delivery of dual A2A/A2B adenosine receptor antagonist M1069 • *A2A* and *A2B* si/shRNA LNP	[357, 356,358]
	L-tryptophan depletion	• Knockout of IDO1 downstream target, aryl hydrocarbon receptor (AhR) • Enhance tryptophanyl tRNA synthetase (TrpRS) expression	• YSK12-C4 multifunctional envelope-type nanodevice for *Ido1* RNAi targeting • cytoplasmic (*WARS*) and mitochondrial *WARS2* mRNA-LNP	[356, 359–361]
Anti-immune response	Lipid peroxidation induced MHCI/II and CD80/CD86 inhibition	• Knockout IRE1α and XBP1 • Enhance NRF2 signaling and expression of GSH and ALDH enzymes	• *ERN1 and XBP1* si/shRNA LNP • *Nrf2* and GSH/ALDH enzyme-encoding mRNA LNPs (engineered proteins)	[97, 362, 356, 363]
	MCT induced antigen processing and presentation inhibition	• Knockout of *MCT1*, *MCT2*, *MCT4*, *SLC5A12* and *SLC5A8*	• *MCT1*, *MCT2*, *MCT4*, *SLC5A12* and *SLC5A8-*encoding siRNA	[86, 356, 364–366]
	Oxysterols- LxRα interaction induced CCR7 downregulation	• Enhance CCR7 and knockout *LxRα*	• CCR7-encoding mRNA LNP • *LxRα* si/shRNA	[98–100, 356]
	ECM remodelling and E-cadherin induced migration inhibition	• Inhibit TGF-β signaling • Knockout lysyl oxidases (LOXs), fibroblast activated proteins (FAPs) and E-cadherin	• *TGF-β-*encoding siRNA LNP • LOX expression RNAi silencing	[356, 367, 368]
	Impaired nucleic acid sensing	• Knockout of *TIM3* • Enhance TLR4 activation	• *TIM3*-encoding siRNA LNPs	[104, 356]
	cGAS/STING pathway downregulation	• Knockout of *Wee1* kinases and *Enpp1*	• *Wee1* and *Enpp1* siRNA LNPs	[108, 369]
	Phagosomal acidification and degradation of tumor mtDNA	• Knockout *Sirpα* and *Shp1* • Enhance NOX2 expression	• sgRNA-CRISPR/Cas9-loaded arginine nanoparticle (ArgNP) • NOX2-encoding mRNA LNP	[106, 370, 371]
Immunosuppression	β-catenin induced cDC1 migration inhibition	• Enhance CCR7 and CXCR3 expression • Promote CD40 agonism and CXCL10 production	• CCR7-encoding mRNA LNP • NP with functionalized CD40 agonist (CD40a)	[110, 113, 371–374]
	VEGF, IL-6 and PGE2 induced inhibition of DC maturation and differentiation	• Enhance CD80/CD86, CD40, CD54, MHCII, CCL19 and IL-12p70 expression	• CD80/CD86-mediated AuNP-induced DC maturation • IL-12-encoding self-replicating RNA-LNP	[60, 112, 237–239, 375–378]
		• Block mIL-6R and IL-6-sIL-6R-Gp130 interaction • Knockout gp130, EP2 and EP4 receptors	• NPs with combinatorial mAb therapy (tocilizumab) • *gp130, EP2,* and *EP4* RNAi targeting	[379–381]
	CTLA-4- CD80/CD86 induced upregulation of IDO1	• *Ido1* and *AhR* knockout • Enhance TrpRS expression	• YSK12-C4 multifunctional envelope-type nanodevice for *Ido1* or *AhR* RNAi targeting	[356, 361, 382]
	Higher Versican-TLR2 interaction	• Block versican interacting domain in TLR2 • Inhibit CD44-hyaluronan-versican interaction • Inhibit IL-6R/IL-10R • Enhance IL-12p70 production	• Blocking of versican-interacting domain and TLR2-MyD88-RelB axis • IL-12-encoding self-replicating RNA-LNP • NPs with *IL-10* siRNA cargo	[378, 383–385]
	Activation of STAT, IL-10, TGF-β and suppression of IL-12	• STAT3 and TGF-β pathway inhibition • Enhance IL-12p70 expression • Knockout IL-10R	• NPs loaded with STAT3 inhibitors (BBI608 and YY002) • IL-12-encoding self-replicating RNA-LNP • Co-delivery of *TGF-β* and *IL-10* siRNA cargo	[7, 378, 385, 386]
	Higher efferocytosis and RTK activation	• Knockout tyrosine protein kinase receptor (TYRO3), AXL and myeloid epithelial receptor tyrosine kinase (MERTK)	• NPs conjugated with TYRO3 neutralizing mAbs • MERTK or AXL extracellular domain ligand sinks/multi-kinase inhibitors (BMS-777607, foretinib, sitravatinib, bosutinib, and vandetinib)	[387–392]
	Upregulation of immune checkpoint molecules	• Knockout *PD-L1*, *VISTA, BTLA* and* LAG-3*	• LNP co-loading of VISTA siRNA and TLR9 agonist (CpG) • Combinatorial therapy (durvalumab, atezolizumab, avelumab) • Glyconanoparticle (GNP) with anti-PD-L1 antibodies	[393–395]

## Data Availability

No datasets were generated or analysed during the current study.

## Abbreviations

ADPGK: ADP-dependent glucokinase
AMP: Adenosine monophosphate
AMPK: AMP-activated protein kinase
APCs: Antigen-presenting cells
AuNCs: Gold nanoclusters
AuNPs: Gold nanoparticles
BLANs: BAMEA-O16B lipid-assisted NPs
cAMP: Cyclic di-adenosine monophosphate
[Ca^2+^]int: Cytosolic calcium
cDCs: Conventional DCs
CD40L: CD40 ligand
CDNs: Cyclic dinucleotides
CCL2: C-C motif Ligand 2
CCL21: C-C motif ligand 21
CCR7: C-C motif chemokine receptor 7
cGAMP: Cyclic guanosine monophosphate-adenosine monophosphate
cGAS: cGAMP synthase
STING: Stimulator of interferon genes
CLAN: Cationic Lipid-Assisted Nanoparticles
Clec9A: C-type lectin domain family 9 member A
CLI: Cerenkov luminescence imaging
CLRs: C-type lectin receptors
CMP: CDN–Mn^2^⁺ particle
CRISPR: Clustered regularly interspaced short palindromic repeats
CRISPRa: CRISPR gene activation approach
CTL: Cytotoxic T lymphocytes
CTLA-4: Cytotoxic T lymphocyte-associated protein 4 antibodies
CX3CL1: C-X3-C motif ligand 1
CXCL9: C-X-C motif chemokine ligand 9
CXCL10: C-X-C motif chemokine ligand 10
CpG: Cytosine (C) phosphate (p) guanine (G) motif
DAMPs: Damage-associated molecular patterns
DCIR2: DC inhibitory receptor-2
DCs: Dendritic cells
DEC-205: Dendritic and epithelial cells-205
dCas9: Dead Cas9
eLNPs: Empty LNPs
ER: Endoplasmic reticulum
EVs: Extracellular vesicles
FAO: Fatty acid oxidation
FcRs: Fc receptors
Fe_3_O_4_: Iron (III) oxide
Flt3L: Fms-like tyrosine kinase 3 ligand
GITR: Glucocorticoid-induced tumor necrosis factor receptor
GM-CSF: Granulocyte-macrophage colony-stimulating factor
HIF-1α: Hypoxia-inducible factor 1α
HLA: Human leukocyte antigen
HMGB1: High mobility group box 1
HSC: Hematopoietic stem cells
HSP70: Heat shock protein 70
ICD: Immunogenic cell death
ICI: Immune checkpoint inhibitor
IDO1: Indoleamine 2,3-dioxygenase 1
IFN-I: Type I interferon
IFN-γ: Interferon gamma
IFNs: Interferons
IL: Interleukin
IRE1α: Inositol-requiring enzyme 1 α
IRE1α-XBP1: Inositol-requiring enzyme 1 α- x-box binding protein 1
IRF3: IFN regulatory factor 3
iPSC: Induced pluripotent stem cell
ISG: Interferon-stimulated gene
LNPs: Lipid nanoparticles
LPO: Lipid peroxidation
LVs: Lentiviral vectors
LXRα: Liver X receptor alpha
MAVS: Mitochondrial antiviral signaling
MDSCs: Myeloid-derived suppressive cells
MHC: Major histocompatibility complex
MITO-TALEN: Mitochondrial-targeted TALEN
MLH1: DNA mismatch repair gene
MRI: Magnetic resonance imaging
MUC1: Mucin 1
MVBs: Multivesicular bodies
MVs: Microvesicles
MYD88: Myeloid differentiation factor 88
moDC: Monocyte-derived DC
mTOR: Mechanistic target of rapamycin
NK: Natural killer
NP: Nanoparticles
ox lipids: Oxidized lipids
OST: Oligosaccharyltransferase
OXPHOS: Oxidative phosphorylation
PAF: Platelet-activating factor
PBMCs: Peripheral blood mononuclear cells
pDC: Plasmacytoid DC
PD-L1: Programmed death ligand 1
PEG: Polyethylene glycol
PEG-b-PLGA: Poly(ethylene glycol)-block-poly(lactide-co-glycolide)
PET: Positron emission tomography
PGE2: Prostaglandin E2
PLG: Poly(lactide-co-glycolide)
PPARγ: Peroxisome proliferator activator receptor gamma
PSA: Prostate-specific antigen
PDT: Photodynamic therapy
PTT: Photothermal therapy
RIe-AuNPs: Radionuclide-embedded AuNPs
RNAi: RNA interference
RNP: Ribonucleoprotein
RTKs: Receptor tyrosine kinases
SIRP-α: CD47-signal regulatory protein-α
TAAs: Tumor-associated antigens
TALENs: Transcription activator-like effector nucleases
TEx: Tumor-derived exosomes
TFH: Follicular T helper
TIDCs: Tumor-infiltrating DCs
TIM3: T cell immunoglobulin mucin receptor 3,
TLR: Toll-like receptor
TME: Tumor microenvironment
TNF: Tumor necrosis factor
TRP2: Tyrosin-related protein 2
TdLN: Tumor-draining lymph node
Treg: Regulatory T cells
UPR: Unfolded protein response
ZFNs: Zinc finger nucleases
ZnO: Zinc oxide
ZnP: Zinc phosphate
